# Activation loop targeting strategy for design of receptor-interacting protein kinase 2 (RIPK2) inhibitors

**DOI:** 10.1016/j.bmcl.2018.01.044

**Published:** 2018-02-15

**Authors:** Chalada Suebsuwong, Daniel M. Pinkas, Soumya S. Ray, Joshua C. Bufton, Bing Dai, Alex N. Bullock, Alexei Degterev, Gregory D. Cuny

**Affiliations:** a Department of Chemistry, University of Houston, Science and Research Building 2, Houston, TX 77204, USA; b Structural Genomics Consortium, University of Oxford, Old Road Campus, Roosevelt Drive, Oxford OX3 7DQ, UK; c Stemetix Inc., 604 Webster St., Needham, MA 02494, USA; d Department of Developmental, Molecular & Chemical Biology, Tufts University School of Medicine, 136 Harrison Avenue, Boston, MA 02111, USA; e Department of Pharmacological and Pharmaceutical Sciences, University of Houston, Science and Research Building 2, Houston, TX 77204, USA

**Keywords:** Kinase, Inhibitor, Activation loop, Receptor-interacting protein kinase 2, RIPK2

## Abstract

Development of selective kinase inhibitors remains a challenge due to considerable amino acid sequence similarity among family members particularly in the ATP binding site. Targeting the activation loop might offer improved inhibitor selectivity since this region of kinases is less conserved. However, the strategy presents difficulties due to activation loop flexibility. Herein, we report the design of receptor-interacting protein kinase 2 (RIPK2) inhibitors based on pan-kinase inhibitor regorafenib that aim to engage basic activation loop residues Lys169 or Arg171. We report development of **CSR35** that displayed >10-fold selective inhibition of RIPK2 versus VEGFR2, the target of regorafenib. A co-crystal structure of **CSR35** with RIPK2 revealed a resolved activation loop with an ionic interaction between the carboxylic acid installed in the inhibitor and the side-chain of Lys169. Our data provides principle feasibility of developing activation loop targeting type II inhibitors as a complementary strategy for achieving improved selectivity.

## Graphical abstract

**Figure:**
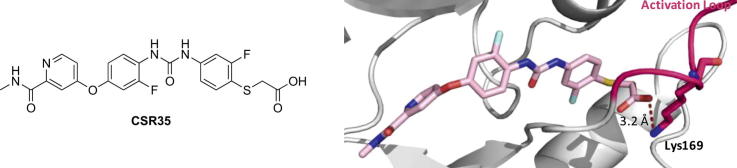

Protein kinases mediate many diverse cellular functions, including gene transcription, metabolism, cell-cycle progression, cytoskeletal rearrangement, movement, proliferation, differentiation and death (e.g. apoptosis and regulated necrosis) by catalyzing phosphate transfer from adenosine triphosphate (ATP) to protein substrates.^1^, ^2^ Dysfunction of many protein kinases is associated with a broad array of human diseases.^3^ Consequently, this class of enzymes has become a key focus for therapeutic development.

The majority of protein kinase inhibitors currently approved for clinical use bind to the catalytically active enzyme at the ATP site, which is located near the hinge region between the N- and C-terminal lobes and are referred to as type I inhibitors.^4^ However, many protein kinases are capable of undergoing conformational changes to catalytically inactive states, which have been targeted with the goal of developing more selective allosteric inhibitors. For example, the αC-helix, the only helical segment in the N-terminal lobe, can rotate to an inactive αC-helix-out conformation.^5^, ^6^ Compounds that bind this state of the enzyme are referred to as type I½ inhibitors.^7^ In another commonly used approach, inhibitors are designed to stabilize kinase conformations in which the three highly conserved amino acids at the beginning of the activation segment (e.g. Asp-Phe-Gly, known as the DFG motif) rotate to the inactive DFG-out state disrupting catalysis. This change also provides access to an adjacent hydrophobic pocket, which inhibitors can bind. Compounds that engage only this hydrophobic pocket are referred to as type III inhibitors, while those that also maintain interactions with the hinge region are termed type II inhibitors.^7^ Over the past two decades, exemplification of these and additional classes of kinase inhibitors has steadily increased with several compounds being approved for clinical use.

A kinase region that has not been widely exploited for inhibitor design is the activation loop. This portion of the kinase is a flexible sub-region of the activation segment and consists of 20–35 amino acid residues that start after the DFG motif and end before the APE, ALE or SPE sequence.^8^, ^9^ The activation loop can mimic the protein substrate by folding and interacting with the substrate binding site. However, in the catalytically active kinase the activation loop is displaced allowing the protein substrates and ATP to bind. The movement of the activation loop is linked to the rotation of the DFG motif from inactive DFG-out to active DFG-in conformations. As a result, the activation loop is “open” (e.g. disengaged) in the active kinase state and oftentimes “closed” (e.g. engaged) in an inactive kinase state.^9^ Designing inhibitors that can form interactions with the activation loop in the “closed” catalytically inactive state can be attractive as sequences and structures of activation segments are poorly conserved. However, this strategy presents a major challenge due to the flexibility of this loop and uncertainty about its precise location and conformation, which is frequently unresolved in reported protein kinase crystal structures. To date, engagement of the activation loop by protein kinase inhibitors has been demonstrated in a small number of cases. For example, the c-Met type I½ inhibitor crizotinib forms a π–π interaction with the side-chain of activation loop residue Y1230 (PDB ID: 2WGJ).^10^ The MEK1 inhibitor PD-184352 forms a series of hydrophobic interactions with the activation loop in addition to a weak F-NH dipole–dipole interaction with the backbone of Ser212 (PDB ID: 1S9J).^11^ Several structurally distinct type III receptor-interacting protein kinase 1 (RIPK1) inhibitors engage activation loop residues through hydrophobic interactions (PDB ID: 4ITI, 4ITJ and 5HX6).^12^, ^13^ Finally, the type III RIPK1 inhibitor Nec-1^14^, ^15^ forms a critical hydrogen bond with the side-chain of activation loop residue Ser161 (PDB ID: 4ITH).^13^ Notably, Nec-1 displays exclusive selectivity for RIPK1 and mutation of Ser161 to Ala was shown to block inhibitor activity.^14^, ^16^ However, in all of these cases it appears that the inhibitor–activation loop interactions were serendipitously discovered and were not part of the original design. Herein, we report our initial efforts at an activation loop targeting design strategy for receptor- interacting protein kinase 2 (RIPK2) by introducing a specific functional group onto pan-specific type II inhibitor regorafenib that we previously discovered to inhibit RIPK2,^17^ leading to improved selectivity against VEGFR2, the primary target of regorafenib. The unique binding mode of the inhibitor engaging the side-chain of activation loop residue Lys169 via an ionic–ionic interaction was confirmed via a RIPK2·inhibitor co-crystal structure.

RIPK2 is a caspase recruitment domain (CARD) containing kinase that has been implicated in nucleotide binding and oligomerization domain 1 and 2 (NOD1 and NOD2) signaling. NOD-RIPK2 interactions result in activation of nuclear factor κB (NF-κB) and mitogen-activated protein (MAP) kinase pathways to promote the transcription of pro-inflammatory cytokines.^18^ This signaling pathway potentially plays a crucial role in an array of pathological conditions including inflammatory bowel disease, Crohn’s disease and multiple sclerosis.^19^

RIPK2 was selected as the target kinase for several reasons. Ponatinib and regorafenib, type II pan-kinase inhibitors that could serve as initial templates for activation loop targeting, have previously been reported to inhibit recombinant RIPK2 with IC_50_ values of 7 and 41 nM, repectively.^17^ A RIPK2 crystal structure in complex with ponatinib (PDB ID: 4C8B) was also available, although the activation loop was unresolved.^17^ Finally, a co-crystal structure of RIPK2 in complex with a biaryl urea inhibitor structurally similar to regorafenib, where part of the activation loop in one of the monomers (chain A) has been resolved, was recently reported (PDB ID: 5AR7).^20^

Regorafenib was selected as the scaffold for activation loop targeting since we previously reported that this molecule more selectively inhibited NOD1/2 signaling in the cells compared to ponatinib.^17^ Molecular docking of regorafenib was initially performed using AutoDockTools-1.5.6 and a RIPK2•ponatinib co-crystal structure lacking a resolved activation loop (PDB ID: 4C8B). The results showed that regorafenib formed similar binding interactions as ponatinib. The regorafenib docked structure was also overlaid with the RIPK2·biaryl urea co-crystal structure that has the activation loop resolved (PDB ID: 5AR7). Again the interactions between the kinase and the two ligands were similar (Fig. 1).

**Fig. 1:**
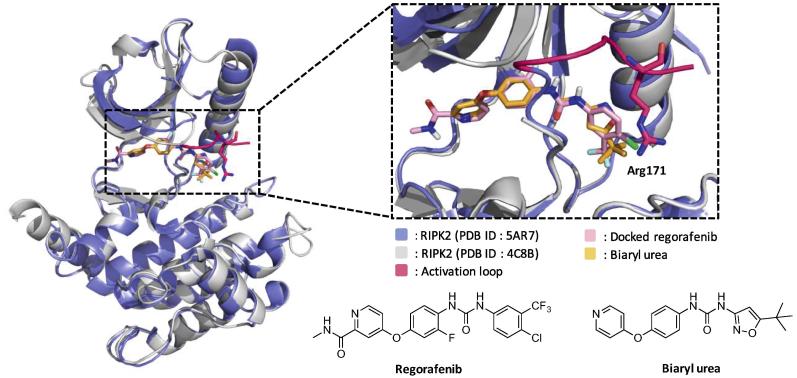
Superimposed crystal structure of docked regorafenib (pink) in RIPK2 lacking a resolved activation loop (gray; PDB ID: 4C8B) and biaryl urea (yellow) in the RIPK2 (blue; PDB ID: 5AR7) structure with a resolved activation loop (highlighted in deep pink).

Next, the kinase domain sequences of RIPK1 and RIPK2 were aligned and compared for regions of divergence. The comparison confirmed alignment of the DXG motifs, where X is Leu in RIPK1 and Phe in RIPK2, at the beginning of the activation segments. Interestingly, RIPK1’s Ser161 residue in the activation loop, which forms the critical hydrogen bond to the RIPK1 inhibitor Nec-1, aligns with Lys169 in RIPK2’s activation loop (Fig. 2A). By contrast, Ser161 in RIPK1 occupied a similar position to Arg171 in RIPK2 when the RIPK1·Nec-1 co-crystal structure was superimposed on the RIPK2·biaryl urea co-crystal structure and the resolved activation loops were compared (Fig. 2B). Since the residues (e.g. Lys169 and Arg171) identified in RIPK2 occupy a similar position as Ser161 in RIPK1 using two independent methods, are both hydrophilic and basic, our design strategy for targeting RIPK2’s activation loop was installation of hydrophilic/acidic functional groups on the urea benzene to enable ionic–dipole or ionic–ionic interactions. Furthermore, the functional groups were installed at the *meta*- and *para*-positions, which were closest to Arg171 (5.5 and 4.4 Å, respectively) based on the docking model of regorafenib with RIPK2 (PDB ID: 5AR7), as shown in Fig. 3. This region of RIPK2 also consists of hydrophobic residues such as Ile69, Leu135 and Leu142. Therefore, more hydrophobic groups, e.g. a *gem*-dimethyl amine, nitrile and ester, were installed for compensating unfavorable interaction with hydrophobic residues in this allosteric pocket. Initially, a synthetically accessible virtual library was generated by using the *in silico* combinatorial library algorithm CombiGlide (Schrödinger LLC) based on regorafenib where the variations were introduced in the urea benzene. According to the docking score and synthetic feasibility, a small library of 15 regorafenib analogs (**CSR** series) with various functional groups on the urea benzene was synthesized in order to introduce ligand–activation loop interactions (Table 1).

**Fig. 2:**
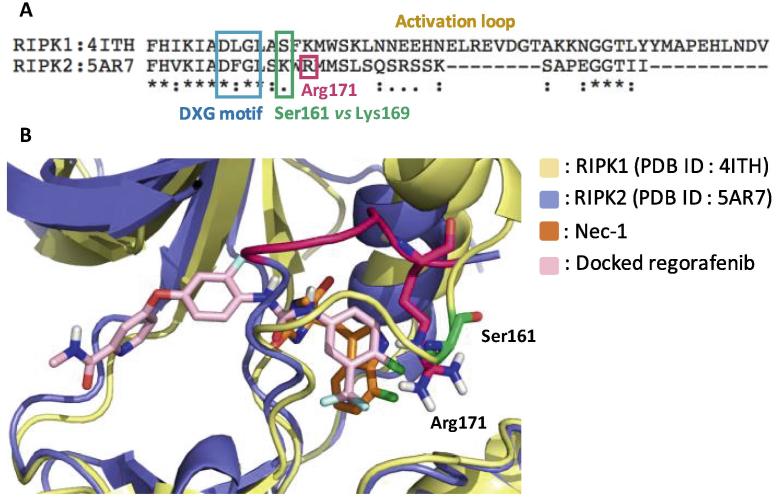
(A) Sequence alignment of the kinase domains of human RIPK1 and RIPK2. The kinase domains were aligned based on sequence similarity (http://www.uniprot.org); (B) Overlay structure showing positions of Ser161 (green) and Arg171 (deep pink) residues in the activation loops of RIPK1 (yellow) and RIPK2 (blue), respectively.

**Fig. 3:**
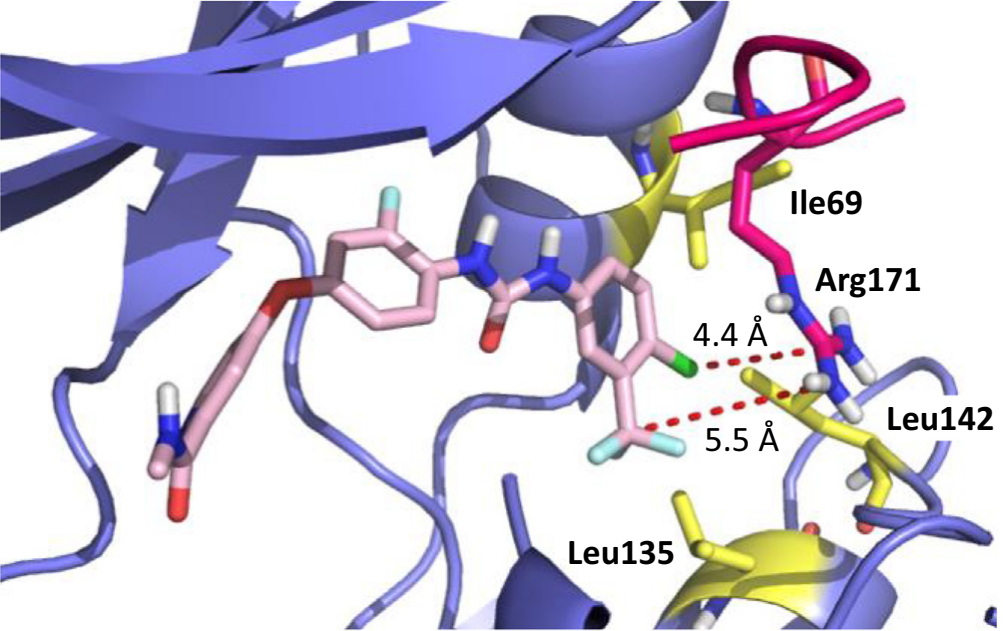
Docking of regorafenib (pink) in RIPK2 (purple; PDB ID: 5AR7) structure with a resolved activation loop (highlighted in deep pink). Hydrophobic residues are highlighted in yellow. Distances from *meta*- and *para*-positions of urea phenyl to Arg171 shown.

**Table 1:** Modifications to the urea benzene targeting the Arg171 residue in the activation loop of RIPK2.

**Figure:**
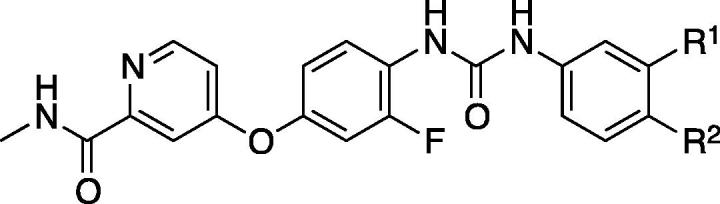

Compound	R^1^	R^2^	Conc. (μM)	% Inhibition	
				RIPK2 WT	R171C RIPK2
**CSR1**	H	COOH	0.5	NI*	ND*
**CSR2**	COOH	H	0.5	NI	ND
**CSR25**		H	0.5	43	ND
**CSR26**	CH_3_		0.5	35	ND
**CSR24**	CH_3_		0.5	1	ND
**CSR27**		H	0.5	12	ND
**CSR28**		H	0.5	32	ND
**CSR31**		H	5.0	NI	NI
**CSR30**		H	5.0	69	76
**CSR29**		H	5.0	47	67
**CSR32**		H	5.0	25	ND
**CSR33**	H		5.0	18	ND
**CSR34**	H		5.0	27	17
**CSR35**	F		5.0	70	64
**CSR36**	F		1.0	94	92

* ND: Not Determined; NI: No Inhibition.

Phenyl urea intermediates with various hydrophilic moieties (**10**) were synthesized by following the methods outlined in Scheme 1, Scheme 2, Scheme 3. To synthesize intermediates **10a**–**d**, a Mitsunobu reaction between nitrophenol **1** and 2-(methylsulfanyl)ethan-1-ol furnished **2**. 2-(3-Nitrophenyl)acetonitrile (**3**) was methylated using iodomethane to give **4**. Hydrolysis of the nitrile under acidic conditions gave carboxylic acid **5**. Esterification of **5** delivered intermediate **6**. Alternatively, **5** was converted to amide **7** using thionyl chloride and ammonium hydroxide. The rearrangement of the primary amide to amine **8** was accomplished using [*I*,*I*-bis(trifluoroacetoxy)iodo]benzene in a mildly acidic mixed of aqueous-organic solvents. The amino group of **8** was protected with Boc to give **9**. The nitrophenyl derivatives **2**, **3**, **6** and **9** underwent iron-mediated nitro reduction to provide **10a**–**d** (Scheme 1).

**Scheme 1:**
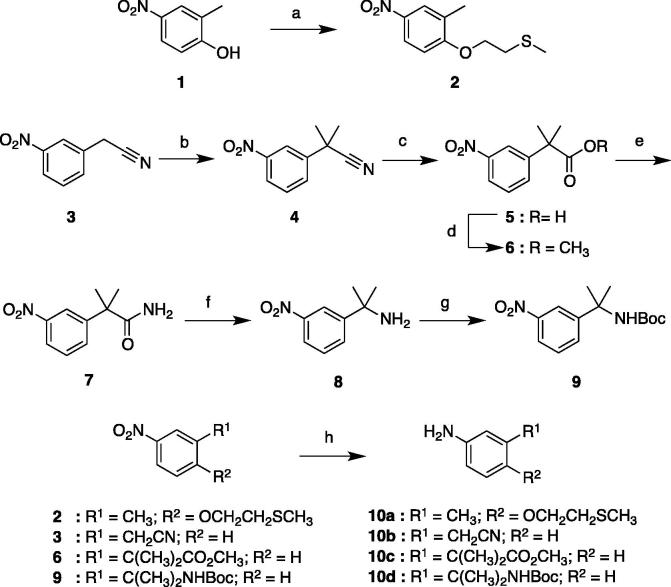
Synthesis of intermediates **10a**–**d**. Reagents and conditions: (a) CH_3_SCH_2_CH_2_OH, DIAD, PPh_3_, THF, 0 °C to rt, 24 h (76%); (b) CH_3_I, NaH, THF, 0 °C to rt, 16 h (30%); (c) H_2_SO_4_, reflux, 16 h (92%); (d) SOCl_2_, MeOH, DME, 0–40 °C, 18 h (78%); (e) i) SOCl_2_, reflux, 16 h, ii) NH_4_OH, 0 °C, 1 h (87%); (f) (F_3_CCO_2_) _2_PhI, H_2_O/MeCN, rt, 18 h (99%); (g) Boc_2_O, NaHCO_3_, THF, 0 °C to rt, 16 h (86%); (h) NH_4_Cl, Fe, EtOH/H_2_O, reflux, 1 h (76–99%).

**Scheme 2:**
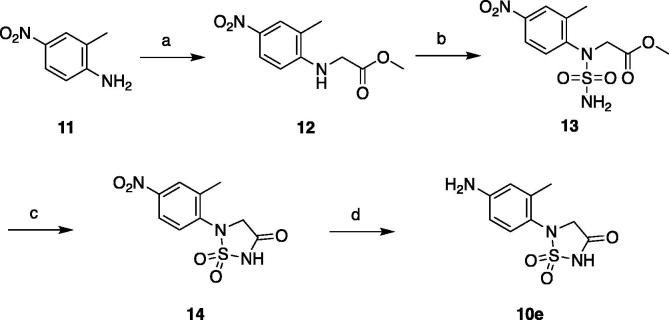
Synthesis of 1,2,5-thiadiazolidin-3-one 1,1-dioxide intermediate **10e**. Reagents and conditions: (a) methyl 2-bromoacetate, Bu_4_NBr, NaHCO_3_, DMF, 90 °C, 18 h (62%); (b) 1) BocNHSO_2_Cl, Et_3_N, CH_2_Cl_2_, 0 °C, 4 h, 2) TFA, CH_2_Cl_2_, rt, 2 h (27% over two steps); (c) NaH, THF, rt, 1 h (96%); (d) NH_4_Cl, Fe, EtOH/H_2_O, reflux, 1 h (81%).

**Scheme 3:**
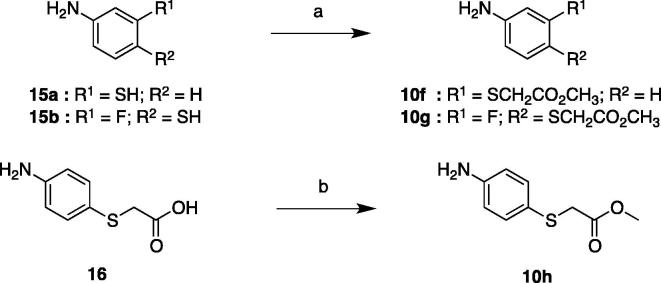
Synthesis of intermediates **10f**–**h**. Reagents and conditions: (a) methyl chloroacetate, K_2_CO_3_, MeCN, rt, 3.5 h (83–99%); (b) SOCl_2_, MeOH, 0 °C to rt, 16 h (93%).

The 1,2,5-thiadiazolidin-3-one 1,1-dioxide intermediate was prepared from commercially available 4-nitro-2-methylaniline (**11**). Substitution of **11** with methyl bromoacetate provided **12**, which was then treated with *tert*-butyl chlorosulfonylcarbamate followed by Boc removal to afford **13**. Cyclization of **13** under basic condition delivered **14**, which was reduced to give aniline **10e** (Scheme 2).

Methyl 2-(phenylthio)acetate intermediates were prepared by either substitution or esterification. Nucleophilic substitution of thiophenols with methyl chloroacetate furnished **10f** and **10g**, while esterification of **16** delivered **10h** (Scheme 3).

**CSR** analogs were synthesized from **10** according to the method outlined in Scheme 4. Nucleophilic aromatic substitution between **17** and 4-amino-3-fluorophenol (**18**) under basic conditions furnished diaryl ether **19**. Intermediates **10a**–**h** or commercially available **10i**–**l** were treated with phenyl chloroformate under basic conditions to provide carbamates **20**. Condensation reactions between **19** and **20** provided **CSR24**–**25**, **30**, **36** and intermediates **21**. Oxidation of **21a** using *m*CPBA furnished **CSR26**. To remove the Boc protecting group, **21d** was treated with TFA to give **CSR28**. Palladium-catalyzed hydrogenation of the nitrile present in **CSR25** delivered primary amine **CSR27**. Methyl ester intermediates were hydrolyzed with lithium hydroxide to yield carboxylic acids **CSR1**–**2**, **29**, and **31**–**35**.

**Scheme 4:**
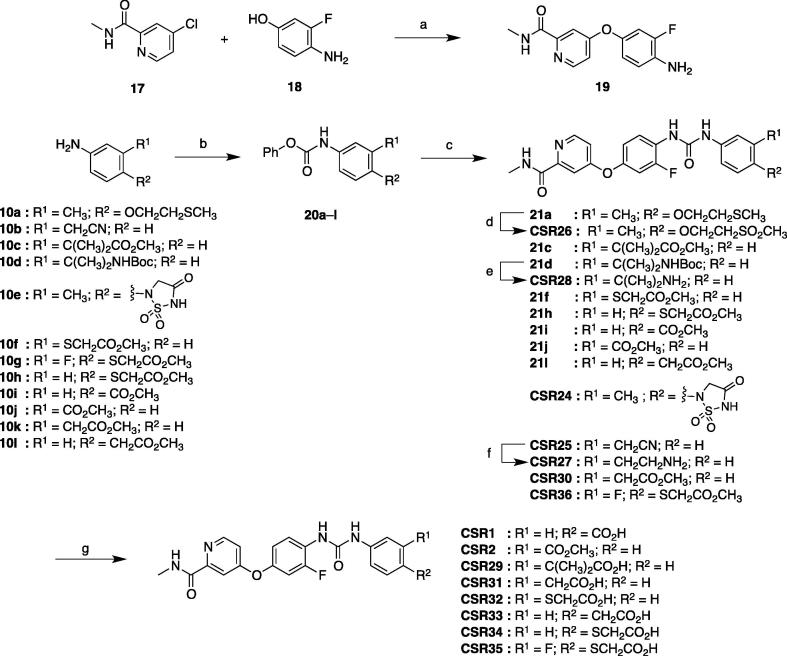
Synthesis of **CSR** analogs with hydrophilic moieties on phenyl ring A. Reagents and conditions: (a) *^t^*BuOK, DMF, rt to 100 °C, 16 h (87%); (b) phenyl chloroformate, Py, CH_2_Cl_2_, 0 °C to rt, 1.5 h (28–99%); (c) **19**, Py, 90 °C, 16 h (28–61%); (d) *m*CPBA, CH_2_Cl_2_, rt, 1 h (31%); (e) TFA, CH_2_Cl_2_, rt, 16 h (84%); (f) H_2_, 10% Pd/C, MeOH, rt, 2 d (99%); (g) LiOH, THF/H_2_O, 60 °C, 18 h (61–98%).

We initially hypothesized that the hydrophilic side-chain might engage Arg171 residue resulting in favorable inhibition of wild-type (WT) RIPK2 compared with R171C RIPK2, where the arginine (from PDB 4C8B) was replaced with cysteine. Therefore, the 15 test compounds were screened for their in vitro RIPK2 enzyme inhibition against RIPK2 WT and the R171C mutant of RIPK2 at a single concentration. One of the carboxylic acid derivatives (e.g. **CSR35**) demonstrated modest percent inhibition in this initial assessment and was selected for further analyses. IC_50_ values of **CSR35** were determined that showed only a twofold preference in RIPK2 WT inhibitory activity (RIPK2 WT IC_50_ = 2.26 ± 0.11 µM versus R171C RIPK2 IC_50_ = 4.87 ± 0.96 µM). Since the carboxylic acid will be deprotonated at pH 7.4, this functional group potentially forms an ionic–ionic interaction with the activation loop. Notably, **CSR35** lacked significant activity in the in vitro enzymatic assay against the primary regorafenib target VEGFR2 (35.8 ± 2.6% inhibition at 20 μM), which does not have a basic residue in the same position as established by a published structure of VEGFR2 in the DFG-out conformation (Fig. 4C).

**Fig. 4:**
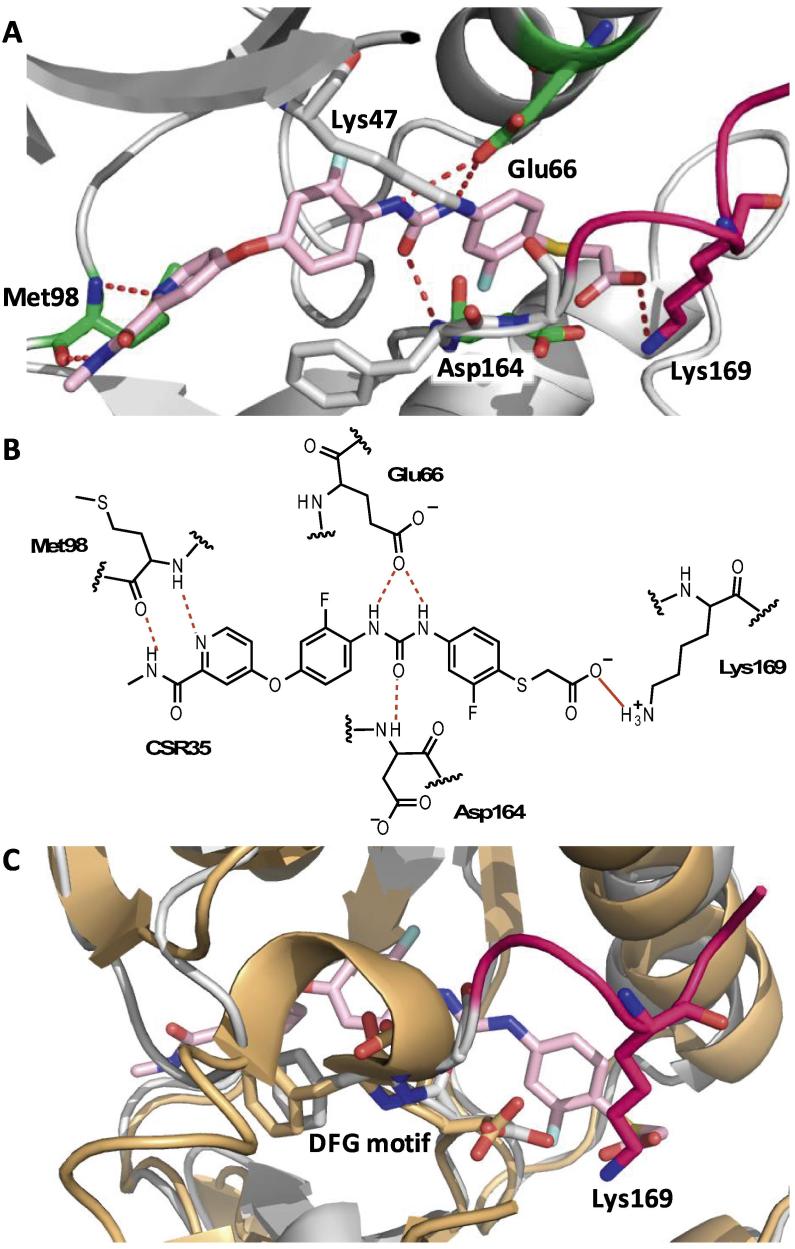
(A and B) Co-crystal structure of **CSR35** (pink) with RIPK2 (gray). Inhibitor **CSR35** forms hydrogen bonds to the backbone of Met98, Glu66 in the αC-helix, and Asp164 in the DFG motif. Lys169 forms an ionic interaction with **CSR35**’s carboxylate side-chain. In addition, a salt bridge between Glu66 on the αC-helix and *β*3-Lys47 is evident in the DFG-out/αC-helix-in conformation. Red dashed lines indicate hydrogen bonds and red solid lines displays the ionic–ionic interactions. (PDB ID: 6ES0); **C**) Superimposed structure of RIPK2·CSR35 (gray and pink) and VEGFR2 (orange; PDB ID: 3WZE).

An X-ray structure of the RIPK2·**CSR35** (PDB ID: 6ES0) was determined at a resolution of 2.4 Å (Fig. 4). Two RIPK2 molecules were found in the asymmetric unit. As expected, **CSR35** was bound to a DFG-out conformation and retained interactions characteristic of most type II inhibitors. The pyridine nitrogen and hydrogen of the amide formed hydrogen bonds with the hinge region (Met98). The urea was engaged in hydrogen bonds with the catalytic residue Glu66 and the backbone NH of Asp164 of the DXG motif. But most interestingly, one of the monomers revealed that Lys169 of the activation loop forms an ionic–ionic interaction with the carboxylate of the ligand and occupied a similar position as Arg171 in the previously reported structure. Engagement of Lys169 was consistent with the predicted result from sequence alignments (Fig. 2A) and likely accounts for modest inhibitory activity differences between WT and R171C mutant RIPK2. In addition, this interaction may be more favorable since protonated amines provide stronger hydrophobic interactions compared to guanidinium ions.^21^ Interestingly, the corresponding methyl ester derivative (**CSR36**) demonstrated potent activity based on the initial screen, raising the possibility that an ionic–dipole interaction with the activation loop might be advantageous (Fig. 5).

**Fig. 5:**
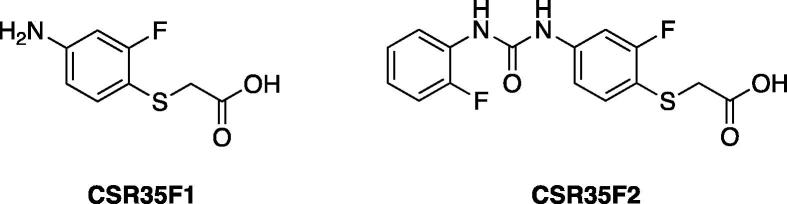
Chemical structure of **CSR35** fragments.

In order to further examine the contributions of the carboxylic acid and urea moieties of **CSR35** in the absence of the hinge binding group two additional compounds were prepared. The first derivative was **CSR35F1** that contained the carboxylic acid, but not the urea. It showed only 10% inhibition at 10 μM. The second derivative (**CSR35F2**) contained both the urea and carboxylic acid. Although it was less potent than **CSR35**, it retained RIPK2 inhibitory activity (IC_50_ = 13.2 ± 1.7 μM). This result raises the possibility that type III inhibitors or type II/III hybrid inhibitors, as was previously generated for RIPK1,^22^ might be feasible for development of RIPK2 inhibitors.

In conclusion, a proof-of-concept for a design strategy of targeting the activation loop of RIPK2 was achieved by introduction of a carboxylic acid fragment into regorafenib. The interactions of **CSR35** with RIPK2, including an ionic–ionic contact with the side-chain of Lys69, were confirmed by X-ray crystallography. Furthermore, a derivative lacking the hinge binding group, which retained modest inhibitory activity, could serve as a scaffold for designing inhibitors (e.g. type III) that engage the activation loop. Finally, given the diversity of activation loop segments among kinases, this strategy may provide an additional means for increasing inhibitor selectively for this class of molecular targets.
