# Supplementary material for: Activation loop targeting strategy for design of receptor-interacting protein kinase 2 (RIPK2) inhibitors
Source: Bioorg Med Chem Lett. 2018 Feb 15;28(4):577–83. doi: 10.1016/j.bmcl.2018.01.044 (PMC5819902; doi:10.1016/j.bmcl.2018.01.044)
Supplement: Supplementary data 1 [file mmc1.docx]

***Supporting Information***

**Activation loop targeting strategy for design of
receptor-interacting protein kinase 2 (RIPK2) inhibitors**

Chalada Suebsuwong^a^, Daniel M. Pinkas^b^, Soumya S. Ray^c^, Joshua C. Bufton^b,d^, Bing Dai^e^, Alex N. Bullock^b^, Alexei Degterev^e^, Gregory D. Cuny^f^

^a^ Department of Chemistry, University of Houston, Science and Research Building 2, Houston, Texas 77204, USA

^b^ Structural Genomics Consortium, University of Oxford, Old Road Campus, Roosevelt Drive, Oxford OX3 7DQ, UK

^c^ Stemetix Inc. 604 Webster St. Needham, MA 02494, USA

^d^ Present address: Department of Biochemistry, University of Bristol, Biomedical Sciences Building, University Walk, Bristol BS8 1TH, UK

^e^ Department of Developmental, Molecular & Chemical Biology, Tufts University School of Medicine, 136 Harrison Avenue, Boston, Massachusetts 02111, USA

^f^ Department of Pharmacological and Pharmaceutical Sciences, University of Houston, Science and Research Building 2, Houston, Texas 77204, USA

**Content**

| 1. General experimental conditions: Chemistry | S2 |
| --- | --- |
| 1. Synthetic procedures | S3-S28 |
| 1. RIPK2 and KDR (VEGFR2) enzyme assays | S28 |
| 1. Table S1. Data collection and refinement statistics for RIPK2•**CSR35** (PDB ID: 6ES0) | S29 |

**General Experimental Conditions: Chemistry**

All reactions involving air-sensitive reagents were carried out with magnetic stirring and in oven-dried glassware with rubber septa under argon unless otherwise stated. All commercially available chemicals and reagent grade solvents were used directly without further purification unless otherwise specified. Reactions were monitored by thin-layer chromatography (TLC) on Baker-flex® silica gel plates (IB2-F) using UV-light (254 and 365 nm) detection or visualizing agents (ninhydrin or phosphomolybdic acid stain). Flash chromatography was conducted on silica gel (230−400 mesh) using Teledyne Isco CombiFlash^®^ Rf. NMR spectra were recorded at room temperature using a JEOL ECA (^1^H NMR at 400, 500, or 600 MHz, and ^13^C NMR at 100, 125, or 150 MHz) with tetramethylsilane (TMS) as an internal standard. Chemical shifts (δ) are given in parts per million (ppm) with reference to solvent signals [^1^H-NMR: CDCl_3_ (7.26 ppm), CD_3_OD (3.30 ppm), DMSO-*d_6_* (2.49 ppm); ^13^C-NMR: CDCl_3_ (77.0 ppm), CD_3_OD (49.0 ppm), DMSO-*d_6_* (39.5 ppm)]. Signal patterns are reported as s (singlet), d (doublet), t (triplet), q (quartet), quin (quintet), sex (sextet), sep (septet), m (multiplet), br (broad), dd (doublet of doublets), dt (doublet of triplets), and td (triplet of doublets). Coupling constants (*J*) are given in Hz. The C-F coupling patterns labeled in ^13^C-NMR indicate visible patterns in spectra. High resolution mass spectra (HRMS) were carried out using AccuTOF (JEOL) spectrometer by the Department of Chemistry, University of Connecticut. The spectra were measured using TOF-MS with a DART or ESI ionization source, and reported as *m/z* (relative intensity) for the molecular ion [M].

**Synthetic Procedures**

**Methyl(2-(2-methyl-4-nitrophenoxy)ethyl)sulfane (2)**

To a mixture of **1** (200.0 mg, 1.31 mmol), triphenylphosphine (311.3 mg, 1.19 mmol), and 2-(methylsulfanyl)ethan-1-ol (0.10 mL, 1.19 mmol) was added anhydrous THF (10 mL) under argon. The reaction was cooled to 0 ºC then diisopropyl azodicarboxylate (0.23 mL, 1.19 mmol) was slowly added. The reaction was stirred at room temperature for 24 h. After being quenched by the addition of water, the aqueous layer was extracted with EtOAc (2 × 15 mL). The combined organic extracts were washed with brine, dried over anhydrous Na_2_SO_4_, filtered, and concentrated. The residue was purified by column chromatography on silica gel (EtOAc/hexane, 5:95 to 20:80) to give **2** (205.0 mg, 76%) as a pale yellow oil; **^1^H NMR** (CDCl_3_, 500 MHz) 8.04 (1 H, dd, J = 8.9, 2.9 Hz), 7.99 (1 H, d, J = 2.9 Hz), 6.82 (1 H, d, J = 9.2 Hz), 4.23 (2 H, t, J = 6.9 Hz), 2.92 (2 H, t, J = 6.3 Hz), 2.25 (3 H, s), 2.21 (3 H, s); **^13^C NMR** (CDCl_3_, 125 MHz) 161.7, 140.9, 127.8, 125.9, 123.4, 109.9, 68.2, 32.8, 16.3, 16.2.

**2-Methyl-2-(3-nitrophenyl)propanenitrile (4)**

To a solution of **3** (400.0 mg, 2.47 mmol) in anhydrous THF (5 mL) was slowly added a suspension of 60% sodium hydride in mineral oil (641.4 mg, 16.03 mmol) in anhydrous THF (4.2 mL) under argon at 0 ºC. Methyl iodide (1.23 mL, 19.74 mmol) was added to the mixture. The temperature was allowed to rise to room temperature and stirred for 16 h. After being quenched by the addition of water, the aqueous layer was extracted with EtOAc (2 × 15 mL). The combined organic extracts were washed with brine, dried over anhydrous Na_2_SO_4_, filtered. The residue was purified by column chromatography on silica gel (EtOAc/hexane, 5:95 to 10:90) to afford **4** (141.3 mg, 30%) as a brown solid; **^1^H NMR** (CDCl_3_, 400 MHz) 8.31 (1 H, t, *J* = 2.3 Hz), 8.21–8.19 (1 H, m), 7.90–7.87 (1 H, m), 7.61 (1 H, t, *J* = 7.8 Hz), 1.79 (6 H, s); **^13^C NMR** (CDCl_3_, 100 MHz) 148.5, 143.5, 131.6, 130.1, 123.3, 123.0, 120.1, 37.1, 28.9.

**2-Methyl-2-(3-nitrophenyl)propanoic acid (5)**

A solution of **5** (60 mg, 0.32 mmol) in 50% H_2_SO_4_ (2 mL) was refluxed for 16 h. The resulting mixture was diluted with EtOAc (15 mL) and water (15 mL). The organic phase was treated with 10% NaOH. The combined aqueous phases were adjusted to pH 2 by 1 N HCl then extracted with EtOAc (2 × 15 mL). The combined organic extracts were washed with brine, dried over anhydrous Na_2_SO_4_, filtered. The solvent was evaporated to give **5** (60.0 mg, 92%) as a brown solid. This product was used in the next step without further purification; **^1^H NMR** (CDCl_3_, 400 MHz) 8.28 (1 H, t, *J* = 1.8 Hz), 8.14 (1 H, dd, *J* = 8.0, 1.4 Hz), 7.74 (1 H, d, *J* = 8.7 Hz), 7.53 (1 H, t, *J* = 8.2 Hz), 1.67 (6 H, s); **^13^C NMR** (CDCl_3_, 100 MHz) 181.8, 148.3, 145.8, 132.5, 129.4, 122.2, 121.1, 46.4, 26.2.

**Methyl 2-methyl-2-(3-nitrophenyl)propanoate (6)**

To a solution of thionyl chloride (0.02 mL, 0.26 mmol) in anhydrous MeOH (0.1 mL) was added a solution of **5** (27.6 mg, 0.13 mmol) in anhydrous DME (0.2 mL) at 0 ºC under argon. The mixture was allowed to rise to room temperature and stirred for 1 h. The temperature was increased to 40 ºC for 18 h. The solvent was removed *in vacuo*, and the residue was purified by column chromatography on silica gel (EtOAc/hexane, 10:90) to give **6** (22.9 mg, 78%) as a yellow oil; **^1^H NMR** (CDCl_3_, 400 MHz) 8.23 (1 H, t, *J* = 1.8 Hz), 8.13–8.10 (1 H, m), 7.68–7.66 (1 H, m), 7.50 (1 H, t, *J* = 7.8 Hz), 3.67 (3 H, s), 1.63 (6 H, s); **^13^C NMR** (CDCl_3_, 100 MHz) 176.1, 148.3, 146.6, 132.3, 129.3, 121.9, 120.9, 52.5, 46.5, 26.4.

**2-Methyl-2-(3-nitrophenyl)propanamide (7)**

A solution of **5** (60.0 mg, 0.29 mmol) in thionyl chloride (2 mL) was refluxed for 16 h. The mixture was allowed to cool to room temperature, and the thionyl chloride was removed *in vacuo.* To the residue was added 6 N ammonium hydroxide (1 mL) and stirred at 0 ºC for 1 h. After being quenched by the addition of water, the aqueous layer was extracted with EtOAc (2 × 15 mL). The combined organic extracts were washed with brine, dried over anhydrous Na_2_SO_4_, filtered. The residue was purified by column chromatography on silica gel (EtOAc/hexane, 20:80 to 80:20) to afford **7** (52.1 mg, 87%) as a brown solid; **^1^H NMR** (CDCl_3_, 400 MHz) 8.28 (1 H, s), 8.14 (1 H, d, *J* = 7.8 Hz), 7.75 (1 H, d, *J* = 6.9 Hz), 7.54 (1 H, t, *J* = 7.8 Hz), 5.62 (1 H, br), 5.31 (1 H, br), 1.64 (6 H, s); **^13^C NMR** (CDCl_3_, 100 MHz) 178.0, 148.2, 147.2, 132.8, 129.6, 122.2, 120.9, 46.9, 26.9.

**2-(3-Nitrophenyl)propan-2-amine (8)**

To a solution of **7** (19.6 mg, 0.09 mmol) in MeCN (1 mL) was added [bis(trifluoroacetoxy)iodo]benzene (40.5 mg, 0.09 mmol) and water (1 mL). The mixture was stirred at room temperature for 18 h. The aqueous phase was added 10% NaOH to adjust to pH 14. After diluting with water, the aqueous layer was extracted with EtOAc (2 × 15 mL). The combined organic extracts were washed with brine, dried over anhydrous Na_2_SO_4_, filtered. The residue was purified by column chromatography on silica gel (MeOH/CH_2_Cl_2_, 10:90 to 20:80) to afford **8** (16.9 mg, 99%) as a yellow oil; **^1^H NMR** (CDCl_3_, 500 MHz) 8.39 (1 H, t, *J* = 1.7 Hz), 8.07 (1 H, dd, *J* = 8.3, 1.7 Hz), 7.87 (1 H, d, *J* = 8.0 Hz), 7.49 (1 H, t, *J* = 8.0 Hz), 2.00 (2 H, br), 1.53 (6 H, s); **^13^C NMR** (CDCl_3_, 125 MHz) 152.2, 148.2, 131.3, 129.1, 121.4, 120.0, 52.6, 32.7.

***tert*-Butyl (2-(3-nitrophenyl)propan-2-yl)carbamate (9)**

To a solution of **8** (16.9 mg, 0.09 mmol) in anhydrous THF (0.7 mL), and saturated aqueous NaHCO_3_ (0.7 mL) was added di-*tert*-butyl dicarbonate (0.06 mL, 0.28 mmol) at 0 ºC. The mixture was allowed to rise to room temperature, and stirred for 16 h. After being quenched by the addition of saturated ammonium chloride solution, the reaction was extracted with EtOAc (2 × 15 mL). The combined organic extracts were washed with brine, dried over anhydrous Na_2_SO_4_, filtered, and concentrated. The residue was purified by column chromatography on silica gel (EtOAc/hexane, 10:90) to afford **9** (23.7 mg, 86%) as a white solid; **^1^H NMR** (CDCl_3_, 500 MHz) 8.26 (1 H, t, *J* = 2.3 Hz), 8.08 (1 H, d, *J* = 8.0 Hz), 7.73 (1 H, d, *J* = 7.4 Hz), 7.49 (1 H, t, *J* = 8.0 Hz), 5.08 (1 H, br), 1.64 (9 H, s), 1.39 (6 H, s); **^13^C NMR** (CDCl_3_, 125 MHz) 154.0, 149.6, 148.3, 131.1, 129.2, 121.5, 120.0, 79.7, 54.7, 29.6, 28.3.

**General Procedure for the Preparation of 3,4-dialkylaniline**; **3-Methyl-4-(2-(methylthio)ethoxy)aniline (10a), Method A.**

To a solution of **2** (150.1 mg, 0.66 mmol) and NH_4_Cl (183.6 mg, 3.43 mmol) in a mixture of EtOH/H_2_O (5:1, 6 mL) was added iron (Fe) powder (184.4 mg, 3.30 mmol) and vigorously stirred at 85 ºC for 1 h. After the mixture was allowed to cool to room temperature, the solid was removed by filtration through a Celite pad, and the filtrate was concentrated. The residue was purified by column chromatography (EtOAc/hexane, 20:70) to afford **10a** (99.2 mg, 76%) as a brown oil; **^1^H NMR** (CDCl_3_, 400 MHz) 6.66 (1 H, d, *J* = 8.7 Hz), 6.54 (1 H, d, *J* = 2.7 Hz), 6.48 (1 H, dd, *J* = 8.5, 3.0 Hz), 4.07 (2 H, t, *J* = 6.9 Hz), 3.27 (1 H, br), 2.86 (2 H, t, *J* = 6.9 Hz), 2.21 (3 H, s), 2.17 (3 H, s); **^13^C NMR** (CDCl_3_, 100 MHz) 149.9, 140.0, 128.2, 118.4, 113.4, 113.0, 68.7, 33.4, 16.3.

**2-(3-Aminophenyl)acetonitrile (10b)**

Method A; purified by column chromatography on silica gel (EtOAc/hexane, 20:80 to 30:70) to give **10b** (98%) as a yellow oil; **^1^H NMR** (CDCl_3_, 500 MHz) 7.12 (1 H, m), 6.65 (1 H, d, *J* = 7.4 Hz), 6.61 (2 H, m), 3.76 (2 H, br), 3.61 (2 H, s); **^13^C NMR** (CDCl_3_, 100 MHz) 147.1, 130.8, 129.8, 118.1, 117.4, 114.3, 113.9, 23.2.

**Methyl 2-(3-aminophenyl)-2-methylpropanoate (10c)**

Method A; purified by column chromatography on silica gel (EtOAc/hexane, 10:90 to 20:80) to give **10c** (99%) as a yellow solid; **^1^H NMR** (CDCl_3_, 500 MHz) 7.11 (1 H, t, *J* = 7.7 Hz), 6.72 (1 H, d, *J* = 7.4 Hz), 6.65 (1 H, s), 6.57 (1 H, d, *J* = 7.4 Hz), 3.65 (3 H, s), 1.54 (6 H, s); **^13^C NMR** (CD_3_OD, 125 MHz) 177.3, 146.4, 145.8, 129.3, 115.8, 113.6, 112.5, 52.2, 46.3, 26.4.

***tert*-Butyl (2-(3-aminophenyl)propan-2-yl)carbamate (10d)**

Method A; purified by column chromatography on silica gel (EtOAc/hexane, 20:80) to give **10d** (99%) as a white solid; **^1^H NMR** (CDCl_3_, 400 MHz) 7.10 (1 H, t, *J* = 8.0 Hz), 6.79 (1 H, d, *J* = 7.8 Hz), 6.73 (1 H, t, *J* = 2.1 Hz), 6.56–6.53 (m, 1H), 4.92 (1 H, br), 3.64 (2 H, br), 1.60 (9 H, s), 1.40 (6 H, s); **^13^C NMR** (CDCl_3_, 100 MHz) 146.2, 129.1, 129.1, 115.2, 113.4, 111.9, 78.8, 54.9, 29.7, 28.3.

**Methyl 2-((2-methyl-4-nitrophenyl)amino)acetate (12)**

To a solution of **11** (30.0 mg, 0.20 mmol) and methyl bromoacetate (0.19 mL, 1.97 mmol) in anhydrous DMF (1.5 mL) was added sodium bicarbonate (331.3 mg, 3.94 mmol) and a catalytic amount of tetrabutylammonium bromide. The mixture was stirred at 90 ºC for 18 h. After being quenched by the addition of water, the aqueous layer was extracted with EtOAc (2 × 15 mL). The combined organic extracts were washed with brine, dried over anhydrous Na_2_SO_4_, filtered, and concentrated. The residue was purified by column chromatography on silica gel (MeOH/CH_2_Cl_2_, 2.5:97.5) to afford **12** (27.2 mg, 62%) as a yellow solid; **^1^H NMR** (CDCl_3_, 400 MHz) 8.05 (1 H, dd, *J* = 8.9, 2.8 Hz), 7.99 (1 H, d, *J* = 2.3 Hz), 6.41 (1 H, d, *J* = 9.2 Hz), 4.94 (1 H, br), 4.03 (2 H, d, *J* = 4.6 Hz), 3.84 (3 H, s), 2.25 (3 H, s); **^13^C NMR** (CDCl_3_, 100 MHz) 170.3, 150.2, 138.2, 126.0, 124.5, 121.8, 108.0, 52.7, 44.8, 17.1.

**Methyl 2-((2-methyl-4-nitrophenyl)(sulfamoyl)amino)acetate (13)**

To a solution of *^t^*BuOH (0.09 mL, 0.85 mmol) in anhydrous CH_2_Cl_2_ (0.5 mL) was added chlorosulfonyl isocyanate (0.09 mL, 0.71 mmol) at 0 ºC. The reaction was stirred for 1 h then the solvent was removed *in vacuo* to generate *tert*-butyl chlorosulfonylcarbamate*.* The *tert*-butyl chlorosulfonylcarbamate in anhydrous CH_2_Cl_2_ (1 mL) and triethylamine (0.08 mL, 0.56 mmol) were added to a solution of **12** (104.7 mg, 0.45 mmol) in anhydrous CH_2_Cl_2_ (2 mL) 0 ºC under argon. The mixture was stirred at 0 ºC for 4 h. After being quenched by the addition of water, the aqueous layer was extracted with CH_2_Cl_2_ (2 × 15 mL). The combined organic extracts were washed with brine, dried over anhydrous Na_2_SO_4_, filtered, and concentrated. The crude product was dissolved in anhydrous CH_2_Cl_2_ (3 mL). Trifluoroacetic acid (0.3 mL) was added to the reaction and stirred for 2 h. After being quenched by the addition of saturated aqueous NaHCO_3_, the aqueous layer was extracted with CH_2_Cl_2_ (2 × 15 mL). The combined organic extracts were washed with brine, dried over anhydrous Na_2_SO_4_, filtered, and concentrated. The residue was purified by column chromatography on silica gel (EtOAc/hexane, 15:85 to 20:80) to afford **13** (38.9 mg, 27%) as a yellow solid; **^1^H NMR** (CDCl_3_, 400 MHz) 8.17 (1 H, d, *J* = 2.8 Hz), 8.06 (1 H, dd, *J* = 8.7, 2.8 Hz), 7.80 (1 H, d, *J* = 8.7 Hz), 5.22 (2 H, s), 3.79 (3 H, s), 2.50 (3 H, s); **^13^C NMR** (CDCl_3_, 100 MHz) 171.4, 147.6, 144.6, 141.5, 130.1, 126.5, 122.1, 54.0, 52.9, 18.5.

**5-(2-Methyl-4-nitrophenyl)-1,2,5-thiadiazolidin-3-one 1,1-dioxide (14)**

To a solution of **13** (76.1 mg, 0.25 mmol) in anhydrous THF (5 mL) was added 60% sodium hydride in mineral oil (13.0 mg, 0.33 mmol) under argon. The reaction was stirred for 1 h at room temperature. After being quenched by the addition of 1 N HCl and water, the aqueous layer was extracted with EtOAc (2 × 15 mL). The combined organic extracts were washed with brine, dried over anhydrous Na_2_SO_4_, filtered. The residue was purified by column chromatography on silica gel (MeOH/CH_2_Cl_2_, 20:80) to afford **14** (65.2 mg, 96%) as a yellow solid; **^1^H NMR** (CD_3_OD, 400 MHz) 8.17 (1 H, d, *J* = 2.3 Hz), 8.08 (1 H, dd, *J* = 8.7, 2.3 Hz), 7.70 (1 H, d, *J* = 8.7 Hz), 4.35 (2 H, s), 2.53 (3 H, s); **^13^C NMR** (CD_3_OD, 100 MHz) 175.6, 148.0, 144.3, 141.9, 130.0, 126.8, 122.9, 59.8, 18.8.

**5-(4-Amino-2-methylphenyl)-1,2,5-thiadiazolidin-3-one 1,1-dioxide (10e)**

Method A; purified by column chromatography on silica gel (MeOH/CH_2_Cl_2_, 20:80) to give **10e** (81%) as a yellow solid; **^1^H NMR** (CD_3_OD, 500 MHz) δ 7.17 (1 H, d, *J* = 8.6 Hz), 6.64 (1 H, d, *J* = 2.3 Hz), 6.60 (1 H, dd, *J* = 8.3, 2.6 Hz), 4.14 (2 H, s), 2.28 (3 H, s); **^13^C NMR** (CD_3_OD, 125 MHz) 177.1, 148.2, 141.0, 130.8, 126.8, 118.4, 115.1, 60.4, 18.4.

**General Procedure for the Preparation of methyl ((aminophenyl)thio)acetates; Methyl 2-((3-aminophenyl)thio)acetate (10f), Method B.**

To a solution of **15a** (0.17 mL, 1.60 mmol) in anhydrous MeCN (16 mL) was added methyl chloroacetate (0.15 mL, 1.76 mmol), and potassium carbonate (242.8 mg, 1.76 mmol) under argon. The mixture was stirred at room temperature for 3.5 h. After being quenched by the addition of water, the aqueous layer was extracted with EtOAc (2 × 15 mL). The combined organic extracts were washed with brine, dried over anhydrous Na_2_SO_4_, filtered, and concentrated. The residue was purified by column chromatography on silica gel (EtOAc/hexane, 30:70) to afford **10e** (320.4 mg, 99%) as a brown oil; **^1^H NMR** (CDCl_3_, 400 MHz) 7.07 (1 H, t, *J* = 7.8 Hz), 6.76–6.74 (1 H, m), 6.72 (1 H, t, *J* = 1.8 Hz), 6.54–6.52 (1 H, m), 3.72 (3 H, s), 3.64 (2 H, s); **^13^C NMR** (CDCl_3_, 100 MHz) 170.3, 146.9, 135.8, 129.8, 119.3, 115.7, 113.7, 52.6, 36.2.

**Methyl 2-((4-amino-2-fluorophenyl)thio)acetate (10g)**

Method B; purified by column chromatography on silica gel (EtOAc/hexane, 20:80 to 50:50) to give **10g** (83%) as a yellow oil; **^1^H NMR** (CDCl_3_, 400 MHz) 7.30–7.25 (1 H, m), 6.42–6.37 (2 H, m), 3.92 (2 H, br), 3.63 (3 H, s), 3.44 (2 H, s); **^13^C NMR** (CDCl_3_, 125 MHz) 170.3, 163.8 (d, *J*_CF_ *=* 244.9 Hz), 149.7 (d, *J*_CF_ = 11.1 Hz), 137.2, 110.9 (d, *J*_CF_ = 2.5 Hz), 106.8 (d, *J*_CF_ = 18.5 Hz), 102.0 (d, *J*_CF_ = 25.8 Hz), 52.2, 37.6.

**Methyl 2-((4-aminophenyl)thio)acetate (10h)**

To a solution of thionyl chloride (0.12 mL, 1.64 mmol) in anhydrous MeOH (0.7 mL) was slowly added a solution of **16** (100.0 mg, 0.55 mmol) in anhydrous MeOH (2 mL) at 0 ºC under argon. The mixture was allowed to rise to room temperature and stirred for 16 h. The solvent was removed *in vacuo*, and the residue was purified by column chromatography on silica gel (EtOAc/hexane, 30:70) to give **10h** (100.0 mg, 93%) as a yellow oil; **^1^H NMR** (CDCl_3_, 500 MHz) 7.28 (2 H, d, *J* = 4.0 Hz), 6.59 (2 H, d, *J* = 8.6 Hz), 3.77 (2 H, br), 3.67 (3 H, s), 3.46 (2 H, s); **^13^C NMR** (CDCl_3_, 125 MHz) 170.6, 146.9, 134.8, 121.4, 115.5, 52.3, 39.0.

**4-(4-Amino-3-fluorophenoxy)-*N*-methylpicolinamide (19)**

To a solution of 4-amino-3-fluorophenol (**18**) (100.0 mg, 0.79 mmol) in anhydrous DMF (3 mL) was added *^t^*BuOK (97.1 mg, 0.86 mmol) under argon. The resulting mixture was stirred at room temperature for 30 min then **17** (120.8 mg, 0.71 mmol) was added. The reaction mixture was stirred at 100 ºC for 16 h. After being quenched with H_2_O (5 mL), the aqueous layer was extracted with EtOAc (2 × 15 mL). The combined organic extracts were washed with brine, dried over anhydrous Na_2_SO_4_, filtered, and concentrated. The residue was purified by column chromatography on silica gel (EtOAc/CH_2_Cl_2_, 5:95 to 20:80) to afford **19** (136 mg, 87%) as a brown solid; **^1^H NMR** (CDCl_3_, 400 MHz) 8.36 (1 H, d, *J* = 5.5 Hz), 7.94 (1 H, br), 7.66 (1 H, d, *J* = 2.8 Hz), 6.93 (1 H, dd, *J* = 5.5, 2.8 Hz), 6.83–6.77 (2 H, m,), 6.73–6.70 (1 H, m), 3.74 (2 H, br), 3.00 (3 H, d, *J* = 5.3 Hz); **^13^C NMR** (CDCl_3_, 100 MHz) 166.6, 164.5, 152.1, 151.2 (d, *J*_CF_ = 241.5 Hz), 149.5, 144.7 (d, *J*_CF_ = 9.6 Hz), 132.6 (d, *J*_CF_ = 12.5 Hz), 117.2 (d, *J*_CF_ *=* 3.8 Hz), 117.0 (d, *J*_CF_ *=* 3.8 Hz), 113.7, 109.6, 109.0 (d, *J*_CF_ = 22.0 Hz), 26.2.

**General Procedure for the Preparation of phenyl phenylcarbamates**; **Phenyl (3-methyl-4-(2-(methylthio)ethoxy)phenyl)carbamate (20a), Method C.**

To a solution of **10a** (88.3 mg, 0.45 mmol) in anhydrous CH_2_Cl_2_ (2 mL) under argon. The reaction was cooled to 0 ºC then phenyl chloroformate (70 μL, 0.54 mmol), and pyridine (40 µL, 0.54 mmol) were slowly added. The reaction was allowed to rise to room temperature and was stirred for 1.5 h. After being quenched by the addition of 1 N HCl and water, the aqueous layer was extracted with EtOAc (2 × 15 mL). The combined organic extracts were washed with brine, dried over anhydrous Na_2_SO_4_, filtered, and concentrated. The residue was purified by column chromatography on silica gel (EtOAc/hexane, 20:80) to give **20a** (96%) as a white oil; **^1^H NMR** (CDCl_3_, 400 MHz) 7.41–7.36 (2 H, m), 7.25–7.17 (5 H, m), 6.84 (1 H, br), 6.77 (1 H, d, *J* = 8.2 Hz), 4.14 (2 H, t, *J* = 6.4 Hz), 2.89 (2 H, t, *J* = 6.4 Hz), 2.26–2.22 (6 H, m); **^13^C NMR** (CDCl_3_, 100 MHz) 153.4, 151.9, 150.6, 130.2, 129.3, 127.8, 125.6, 122.1, 121.6, 117.5, 111.6, 68.0, 33.2, 16.4, 16.3.

**Phenyl (3-(cyanomethyl)phenyl)carbamate (10b)**

Method C; purified by column chromatography on silica gel (EtOAc/hexane, 30:70 to 40:60) to give **10b** (95%) as a white oil; **^1^H NMR** (CDCl_3_, 500 MHz) 7.50–7.40 (2 H, m), 7.36–7.31 (3 H, m), 7.24–7.19 (2 H, m), 7.14 (2 H, d, *J* = 8.0 Hz), 6.99 (1 H, d, *J* = 7.4 Hz), 3.60 (2 H, s); 3.70 (2 H, br), 1.37 (3 H, t, *J* = 6.9 Hz); **^13^C NMR** (CDCl_3_, 125 MHz) 151.8, 150.2, 138.2, 130.7, 129.7, 129.3, 125.7, 123.0, 121.5, 118.2, 118.0, 117.8, 23.3.

**Methyl 2-methyl-2-(3-((phenoxycarbonyl)amino)phenyl)propanoate (20c)**

Method C; purified by column chromatography on silica gel (EtOAc/hexane, 10:90) to give **20c** (96%) as a white solid; **^1^H NMR** (CDCl_3_, 500 MHz) 7.47 (1 H, s), 7.40–7.33 (3 H, m), 7.29–7.22 (2 H, m), 7.18 (2 H, d, *J* = 7.4 Hz), 7.13 (1 H, br), 7.06 (1 H, d, *J* = 7.4 Hz), 3.65 (3 H, s), 1.57 (6 H, s); **^13^C NMR** (CDCl_3_, 125 MHz) 177.1, 151.6, 150.4, 145.8, 137.5, 129.4, 129.1, 125.7, 121.6, 121.2, 117.1, 116.1, 52.3, 46.5, 26.4.

***tert*-Butyl (2-(3-((phenoxycarbonyl)amino)phenyl)propan-2-yl)carbamate (20d)**

Method C; purified by column chromatography on silica gel (EtOAc/hexane, 20:80) to give **20d** (92%) as a brown solid; **^1^H NMR** (CDCl_3_, 500 MHz) 7.56 (1 H, br), 7.39 (2 H, t, *J* = 7.4 Hz), 7.28–7.22 (3 H, m), 7.19–7.14 (4 H, m), 4.98 (1 H, s), 1.61 (9 H, s), 1.40 (6 H, s); **^13^C NMR** (CDCl_3_, 125 MHz) 154.0, 151.7, 150.5, 148.8, 137.4, 129.4, 128.9, 125.6, 121.7, 120.3, 116.7, 115.4, 79.2, 54.8, 29.4, 28.3.

**Phenyl (4-(1,1-dioxido-4-oxo-1,2,5-thiadiazolidin-2-yl)-3-methylphenyl)carbamate (20e)**

Method C; purified by column chromatography on silica gel (MeOH/CH_2_Cl_2_, 20:80) to give **20e** (42%) as a white solid; **^1^H NMR** (CD_3_OD, 500 MHz) 7.42–7.38 (5 H, m), 7.23 (1 H, t, *J* = 6.8 Hz), 7.18 (2 H, d, *J* = 7.4 Hz), 4.20 (2 H, s), 2.39 (3 H, s); **^13^C NMR** (CD_3_OD, 125 MHz) 176.9, 154.0, 152.3, 141.1, 139.7, 132.1, 130.5, 130.4, 126.6, 122.9, 121.8, 118.2, 60.4, 18.6.

**Methyl 2-((3-((phenoxycarbonyl)amino)phenyl)thio)acetate (20f)**

Method C; purified by column chromatography on silica gel (EtOAc/hexane, 10:90 to 20:80) to give **20f** (96%) as a clear oil; **^1^H NMR** (CDCl_3_, 400 MHz) 7.56 (1 H, s), 7.42–7.38 (2 H, m), 7.31–7.22 (4 H, m), 7.20–7.17 (2 H, m), 7.14–7.11 (1 H, m), 3.73 (3 H, s), 3.68 (2 H, s); **^13^C NMR** (CDCl_3_, 100 MHz) 170.1, 151.3, 150.4, 138.1, 136.2, 129.6, 129.4, 125.8, 124.6, 121.6, 119.2, 117.0, 52.7, 36.0.

**Methyl 2-((2-fluoro-4-((phenoxycarbonyl)amino)phenyl)thio)acetate (20g)**

Method C; purified by column chromatography on silica gel (EtOAc/hexane, 10:90 to 25:75) to give **20g** (90%) as a white oil; **^1^H NMR** (CDCl_3_, 500 MHz) 7.46–7.39 (4 H, m), 7.28–7.22 (2 H, m), 7.18 (2 H, dd, *J* = 8.6, 1.2 Hz), 7.07 (1 H, dd, *J* = 8.6, 2.3 Hz), 3.70 (3 H, s), 3.56 (2 H, s); **^13^C NMR** (CDCl_3_, 125 MHz) 170.1, 162.7 (d, *J*_CF_ = 246.1 Hz), 151.2, 150.2, 139.7 (d, *J*_CF_ = 9.8 Hz), 135.4, 129.5, 126.0, 121.5, 121.1, 114.3, 106.5 (d, *J*_CF_ = 30.8 Hz), 52.3, 36.5.

**Methyl 2-((4-((phenoxycarbonyl)amino)phenyl)thio)acetate (20h)**

Method C; purified by column chromatography on silica gel (EtOAc/hexane, 10:90 to 20:80) to give **20h** (91%) as a white solid; **^1^H NMR** (CDCl_3_, 500 MHz) 7.41–7.38 (6 H, m), 7.26–7.23 (1 H, m), 7.19–7.17 (2 H, m), 7.12 (1 H, br), 3.71 (3 H, s), 3.59 (2 H, s); **^13^C NMR** (CDCl_3_, 125 MHz) 170.3, 151.4, 150.4, 137.0, 132.3, 129.4, 129.0, 125.8, 121.6, 119.2, 52.5, 37.5.

**Methyl 4-((phenoxycarbonyl)amino)benzoate (20i)**

Method C; purified by column chromatography on silica gel (MeOH/CH_2_Cl_2_, 2:98) to give **20i** (99%) as a white solid; **^1^H NMR** (CDCl_3_, 400 MHz) 8.04–8.00 (2 H, m), 7.52 (2 H, d, *J* = 8.7 Hz), 7.43–7.39 (2 H, m), 7.28–7.24 (1 H, m), 7.20–7.17 (2 H, m), 3.91 (3 H, s); **^13^C NMR** (CDCl_3_, 100 MHz) 166.6, 151.3, 150.2, 141.6, 131.0, 129.5, 126.0, 125.3, 121.5, 117.8, 52.0.

**Methyl 3-((phenoxycarbonyl)amino)benzoate (20j)**

Method C; purified by column chromatography on silica gel (MeOH/CH_2_Cl_2_, 2:98) to give **20j** (99%) as a white solid; **^1^H NMR** (CDCl_3_, 400 MHz) 8.12 (1 H, s), 7.85 (1 H, d, *J* = 7.8 Hz), 7.77–7.74 (2 H, m), 7.38 (3 H, t, *J* = 8.2 Hz), 7.25–7.21 (1 H, m), 7.17 (2 H, d, *J* = 7.8 Hz), 3.91 (3 H, s); **^13^C NMR** (CDCl_3_, 100 MHz) 166.9, 151.8, 150.4, 138.0, 130.7, 129.4, 129.2, 125.7, 124.6, 123.0, 121.6, 119.8, 52.3.

**Methyl 2-(3-((phenoxycarbonyl)amino)phenyl)acetate (20k)**

Method C; purified by column chromatography on silica gel (EtOAc/hexane, 10:90 to 15:85) to give **20k** (99%) as a yellow oil; **^1^H NMR** (CDCl_3_, 400 MHz) 7.28–7.30 (5 H, m), 7.25–7.20 (2 H, m), 7.15 (2 H, d, *J* = 7.8 Hz), 6.99 (1 H, d, *J* = 7.3 Hz), 3.68 (3 H, s), 3.59 (2 H, s); **^13^C NMR** (CDCl_3_, 100 MHz) 172.0, 151.6, 150.4, 137.7, 134.8, 129.3, 129.2, 125.6, 124.6, 121.6, 119.5, 117.4, 52.1, 41.0.

**Methyl 2-(4-((phenoxycarbonyl)amino)phenyl)acetate (20l)**

Method C; purified by column chromatography on silica gel (EtOAc/hexane, 20:80) to give **20l** (96%) as a white solid; **^1^H NMR** (CDCl_3_, 500 MHz) 7.41–7.37 (4 H, m), 7.25–7.22 (3 H, m), 7.19–7.17 (2 H, m), 6.97 (1 H, br), 3.68 (3 H, s), 3.59 (2 H, s); **^13^C NMR** (CDCl_3_, 125 MHz) 172.0, 151.5, 151.2, 150.5, 136.4, 130.0, 129.4, 125.7, 121.6, 118.8, 52.1, 40.5.

**General Procedure for the Preparation of 6-(3-fluoro-4-(3-alkylphenylureido)phenoxy)-*N*-methylpyrimidine-4-carboxamides; 4-(3-Fluoro-4-(3-(3-methyl-4-(2-(methylthio)ethoxy)phenyl)ureido)phenoxy)-*N*-methylpicolinamide (21a), Method D.**

To a mixture of **20a** (87.5 mg, 0.28 mmol) and **19** (55.4 mg, 0.21 mmol) was added pyridine (2.0 mL) under argon. The reaction was put into a pre-heated oil bath (90 ºC) and stirred for 16 h. After being quenched by the addition of 1 N HCl and water, the aqueous layer was extracted with EtOAc (2 × 15 mL). The combined organic extracts were washed with brine, dried over anhydrous Na_2_SO_4_, filtered, and concentrated. The residue was purified by column chromatography on silica gel (EtOAc/CH_2_Cl_2_, 30:70) to afford **21a** (47.3 mg, 46%) as a yellow solid; **^1^H NMR** (CDCl_3_, 500 MHz) 8.39 (1 H, d, *J* = 5.7 Hz), 8.33–8.23 (3 H, m), 7.93 (1 H, br), 7.63 (1 H, d, *J* = 2.3 Hz), 7.17–7.14 (2 H, m), 7.03 (1 H, dd, *J* = 5.4, 2.3 Hz), 6.83–6.78 (2 H, m), 6.70 (1 H, d, *J* = 8.6 Hz), 4.08 (2 H, t, *J* = 6.3 Hz), 3.00 (3 H, d, *J* = 5.2 Hz), 2.85 (2 H, t, *J* = 6.9 Hz), 2.20 (3 H, s), 2.15 (3 H, s); **^13^C NMR** (CDCl_3_, 125 MHz) 166.2, 165.2, 153.5, 153.2, 152.2 (d, *J*_CF_ = 246.1 Hz), 151.5, 149.8, 147.3 (d, *J*_CF_ = 9.8 Hz), 131.2, 127.5, 126.0 (d, *J*_CF_ = 9.8 Hz), 123.9, 121.8, 119.3, 116.8, 114.9, 111.7, 109.2, 108.2 (d, *J*_CF_ = 20.9 Hz), 68.0, 33.2, 26.4, 16.3, 16.2.

**4-(3-Fluoro-4-(3-(3-methyl-4-(2-(methylsulfonyl)ethoxy)phenyl)ureido)phenoxy)-*N*-methylpicolinamide (CSR26)**

To a solution of **21a** (23.4 mg, 0.05 mmol) in anhydrous CH_2_Cl_2_ (2 mL) was added a solution of 3-chloroperbenzoic acid (33.3 mg, 0.10 mmol) in anhydrous CH_2_Cl_2_ (0.45 mL). The mixture was stirred at room temperature for 1 h. After being quenched with addition of saturated aqueous NaHCO_3_ (5 mL), CH_2_Cl_2_ were added, and the layers were separated. The combined organic phases were washed with brine, dried over anhydrous Na_2_SO_4_, filtered, and concentrated. The residue was purified by column chromatography on silica gel (MeOH/CH_2_Cl_2_, 5:95 to 20:80) to give **CSR26** (5.4 mg, 31%) as a yellow solid; **^1^H NMR** (CDCl_3_, 500 MHz) 8.42 (1 H, d, *J* = 5.7 Hz), 8.35 (1 H, t, *J* = 8.6 Hz), 8.27–8.26 (2 H, m), 7.85 (1 H, s), 7.61 (1 H, d, *J* = 1.7 Hz), 7.18 (1 H, d, *J* = 8.6 Hz), 7.10 (1 H, dd, *J* = 5.2, 2.3 Hz), 6.87–6.80 (2 H, m), 6.75 (1 H, d, *J* = 8.6 Hz), 4.38 (2 H, t, *J* = 5.2 Hz), 3.45 (2 H, t, *J* = 5.2 Hz), 3.08 (3 H, s), 3.01 (3 H, d, *J* = 4.6 Hz), 2.17 (3 H, s); **^13^C NMR** (CDCl_3_, 125 MHz) 166.3, 165.2, 153.2, 152.1 (d, *J*_CF_ = 244.9 Hz), 152.0, 151.5, 149.8, 147.3 (d, *J*_CF_ = 9.8 Hz), 132.4, 127.2, 126.0 (d, *J*_CF_ = 9.8 Hz), 123.8, 121.6, 119.1, 117.0, 115.2, 111.8, 108.9, 108.3 (d, *J*_CF_ = 22.2 Hz), 62.4, 54.9, 43.0, 26.4, 16.5.

**Methyl 2-(3-(3-(2-fluoro-4-((2-(methylcarbamoyl)pyridin-4-yl)oxy)phenyl)ureido)phenyl)-2-methylpropanoate (21c)**

Method D; purified by column chromatography on silica gel (EtOAc/CH_2_Cl_2_, 5:95 to 25:75) to afford **21c** (54%) as a white solid; **^1^H NMR** (CDCl_3_, 600 MHz) 8.58 (1 H, s), 8.43 (1 H, d, *J* = 5.5 Hz), 8.36 (1 H, t, *J* = 9.6 Hz), 8.31 (1 H, d, *J* = 4.8 Hz), 7.97 (1 H, s), 7.62 (1 H, d, *J* = 2.8 Hz), 7.46 (1 H, s), 7.34 (1 H, d, *J* = 8.2 Hz), 7.22 (1 H, t, *J* = 8.2 Hz), 7.11 (1 H, dd, *J* = 5.5, 2.1 Hz), 6.98 (1 H, d, *J* = 7.6 Hz), 6.86 (1 H, d, *J* = 8.9 Hz), 6.80 (1 H, dd, *J* = 11.0, 2.1 Hz), 3.64 (3 H, s), 3.03 (3 H, d, *J* = 4.8 Hz), 1.55 (6 H, s); **^13^C NMR** (CDCl_3_, 150 MHz) 177.5, 166.4, 165.3, 152.8, 152.2 (d, *J*_CF_ = 245.4 Hz), 151.4, 149.8, 147.2 (d, *J*_CF_ = 8.9 Hz), 145.4, 139.0, 128.9, 126.0 (d, *J*_CF_ = 10.4 Hz), 121.8, 120.3, 118.0, 117.0, 116.8, 115.2, 108.9, 108.2 (d, *J*_CF_ = 22.2 Hz), 52.3, 46.5, 29.7, 26.4.

**tert-butyl (2-(3-(3-(2-fluoro-4-((2-(methylcarbamoyl)pyridin-4-yl)oxy)phenyl)ureido)phenyl)propan-2-yl)carbamate (21d)**

Method D; purified by column chromatography on silica gel (EtOAc/CH_2_Cl_2_, 5:95 to 30:70) to afford **21d** (51%) as a white solid; **^1^H NMR** (CDCl_3_, 500 MHz) 8.40 (1 H, d, *J* = 5.7 Hz), 8.28 (1 H, t, *J* = 9.2 Hz), 8.13 (1 H, s), 7.85 (1 H, s), 7.78 (1 H, s), 7.68–7.65 (2 H, m), 7.13 (1 H, s), 7.05 (1 H, d, *J* = 7.4 Hz), 7.01 (1 H, s), 6.85–6.80 (2 H, m), 6.72 (1 H, s), 5.24 (1 H, s), 3.02 (3 H, d, *J* = 5.2 Hz), 1.63 (6 H, s), 1.44 (9 H, s); **^13^C NMR** (CDCl_3_, 125 MHz) 166.3, 164.8, 155.2, 152.6, 152.4 (d, *J*_CF_ = 246.1 Hz), 151.4, 149.8, 147.4 (d, *J*_CF_ = 9.8 Hz), 139.1, 128.6, 125.9 (d, *J*_CF_ = 9.8 Hz), 122.0, 119.0, 117.6, 116.8, 115.9, 114.3, 113.9, 109.6, 108.3 (d, *J*_CF_ = 22.2 Hz), 79.9, 54.9, 29.7, 28.3, 26.2.

**4-(4-(3-(3-(2-aminopropan-2-yl)phenyl)ureido)-3-fluorophenoxy)-*N*-methylpicolinamide (CSR28)**

To a solution of **21d** (21.0 mg, 0.04 mmol) in anhydrous CH_2_Cl_2_ (2 mL) was added trifluoroacetic acid (0.2 mL). The mixture was stirred at room temperature for 16 h. After being quenched with saturated aqueous NaHCO_3_ (5 mL), CH_2_Cl_2_ were added, and the layers were separated. The combined organic phases were washed with brine, dried over anhydrous Na_2_SO_4_, filtered, and concentrated. The residue was purified by column chromatography on silica gel (EtOAc/CH_2_Cl_2_, 20:80 to 30:70) to give **CSR28** (20.8 mg, 84%) as a yellow solid; **^1^H NMR** (DMSO-*d_6_*, 500 MHz) 9.22 (1 H, s), 8.80 (1 H, d, *J* = 4.6 Hz), 8.69 (1 H, s), 8.52 (1 H, d, *J* = 5.2 Hz), 8.22 (1 H, t, *J* = 9.2 Hz), 7.60 (1 H, s), 7.40–7.27 (4 H, m), 7.19–7.15 (2 H, m), 7.05 (1 H, d, *J* = 9.2 Hz), 2.78 (3 H, d, *J* = 4.6 Hz), 1.48 (6 H, s); **^13^C NMR** (DMSO-*d_6_*, 125 MHz) 165.6, 163.7, 152.5, 152.3 (d, *J*_CF_ = 247.3 Hz), 152.2, 150.5, 147.4 (d, *J*_CF_ = 9.8 Hz), 139.3, 128.8, 125.5 (d, *J*_CF_ = 11.1 Hz), 121.8, 118.9, 117.1, 116.7, 114.9, 114.1, 109.1 (d, *J*_CF_ = 24.6 Hz), 108.0, 53.6, 30.3, 26.0.

**Methyl 2-((3-(3-(2-fluoro-4-((2-(methylcarbamoyl)pyridin-4-yl)oxy)phenyl)ureido)phenyl)thio)acetate (21f)**

Method D; purified by column chromatography on silica gel (EtOAc/CH_2_Cl_2_, 5:95 to 25:75) to afford **21f** (31%) as a white solid; **^1^H NMR** (CDCl_3_, 500 MHz) 8.66 (1 H, s), 8.43 (1 H, d, *J* = 5.2 Hz), 8.31–8.28 (2 H, m), 7.97 (1 H, s), 7.61 (1 H, s), 7.58 (1 H, d, *J* = 2.3 Hz), 7.23–7.22 (1 H, m), 7.17 (1 H, t, *J* = 8.0 Hz), 7.11 (1 H, dd, *J* = 5.4, 2.3 Hz), 7.01 (1 H, d, *J* = 7.4 Hz), 6.84 (1 H, d, *J* = 9.2 Hz), 6.78 (1 H, dd, *J* = 11.2, 2.3 Hz), 3.72 (3 H, s), 3.68 (2 H, s), 3.02 (3 H, d, *J* = 5.2 Hz); **^13^C NMR** (CDCl_3_, 125 MHz) 170.6, 166.3, 165.3, 152.6, 152.2 (d, *J*_CF_ = 244.9 Hz), 151.3, 149.9, 139.7, 135.5, 129.4, 127.8, 125.8 (d, *J*_CF_ = 9.8 Hz), 123.5, 121.7, 119.6, 117.6, 117.0 (d, *J*_CF_ = 2.5 Hz), 115.2, 108.8, 108.2 (d, *J*_CF_ = 22.2 Hz), 52.7, 36.2, 26.5.

**Methyl 2-((4-(3-(2-fluoro-4-((2-(methylcarbamoyl)pyridin-4-yl)oxy)phenyl)ureido)phenyl)thio)acetate (21h)**

Method D; purified by column chromatography on silica gel (EtOAc/CH_2_Cl_2_, 5:95 to 25:75) to afford **21h** (44%) as a brown solid; **^1^H NMR** (CDCl_3_, 400 MHz) 8.77 (1 H, s), 8.44 (1 H, d, *J* = 5.5 Hz), 8.38–8.33 (2 H, m), 8.06 (1 H, s), 7.58 (1 H, d, *J* = 2.3 Hz), 7.42–7.34 (4 H, m), 7.13 (1 H, dd, *J* = 5.3, 2.3 Hz), 6.86 (1 H, d, *J* = 9.2 Hz), 6.79 (1 H, dd, *J* = 11.2, 2.3 Hz), 3.69 (3 H, s), 3.57 (2 H, s), 3.02 (3 H, d, *J* = 5.0 Hz); **^13^C NMR** (CDCl_3_, 100 MHz) 170.6, 166.3, 165.4, 152.6, 152.0 (d, *J*_CF_ = 245.5 Hz), 151.2, 149.9, 147.1 (d, *J*_CF_ = 9.8 Hz), 138.9, 132.5, 127.2, 125.9 (d, *J*_CF_ = 9.8 Hz), 121.6, 119.7, 117.0,115.4, 108.6, 108.2 (d, *J*_CF_ = 21.5 Hz), 52.5, 37.8, 26.5.

**Methyl 4-(3-(2-fluoro-4-((2-(methylcarbamoyl)pyridin-4-yl)oxy)phenyl)ureido)benzoate (21i)**

Method D; purified by column chromatography on silica gel (MeOH/CH_2_Cl_2_, 2:98 to 5:95) to afford **21i** (28%) as a white solid; **^1^H NMR** (CDCl_3_, 400 MHz) 9.11 (1 H, s), 8.46 (1 H, d, *J* = 5.5 Hz), 8.44–8.42 (2 H, m), 8.21 (1 H, d, *J* = 2.8 Hz), 7.96 (2 H, d, *J* = 8.7 Hz), 7.58–7.54 (3 H, m), 7.18 (1 H, dd, *J* = 5.5, 2.8 Hz), 6.91–6.88 (1 H, m), 6.81 (1 H, dd, *J* = 11.4, 2.8 Hz), 3.88 (3 H, s), 3.03 (3 H, d, *J* = 5.0 Hz); **^13^C NMR** (CDCl_3_, 100 MHz) 167.0, 166.4, 165.6, 152.3, 151.9 (d, *J*_CF_ = 258.8 Hz), 151.1, 149.9, 147.1 (d, *J*_CF_ = 9.6 Hz), 130.8, 126.0 (d, *J*_CF_ = 9.6 Hz), 125.5, 123.8, 121.4, 117.8, 117.2, 115.8, 108.4, 108.1 (d, *J*_CF_ = 24.0 Hz), 51.9, 26.6.

**Methyl 3-(3-(2-fluoro-4-((2-(methylcarbamoyl)pyridin-4-yl)oxy)phenyl)ureido)benzoate (21j)**

Method D; purified by column chromatography on silica gel (EtOAc/CH_2_Cl_2_, 5:95 to 25:75) to afford **21j** (49%) as a white solid; **^1^H NMR** (CDCl_3_, 400 MHz) 8.96 (1 H, s), 8.45–8.36 (3 H, m), 8.17 (1 H, d, *J* = 2.8 Hz), 8.04 (1 H, s), 7.80 (1 H, dd, *J* = 8.0, 1.4 Hz), 7.68 (1 H, d, *J* = 7.8 Hz), 7.60 (1 H, d, *J* = 2.3 Hz), 7.34 (1 H, t, *J* = 7.8 Hz), 7.14 (1 H, dd, *J* = 5.5, 2.8 Hz), 6.87 (1 H, dd, *J* = 9.2, 1.4 Hz), 6.80 (1 H, dd, *J* = 11.4, 2.8 Hz), 3.87 (3 H, s), 3.04 (3 H, d, *J* = 5.0 Hz); **^13^C NMR** (CDCl_3_, 100 MHz) 167.1, 166.3, 165.5, 152.8, 152.0 (d, *J*_CF_ = 246.3 Hz), 151.2, 149.9, 147.1 (d, *J*_CF_ = 9.6 Hz), 139.3, 130.7, 129.0, 125.9, 123.8 (d, *J*_CF_ = 12.5 Hz), 121.6, 120.0, 117.1, 117.0, 115.5, 108.6, 108.2 (d, *J*_CF_ = 21.1 Hz), 52.1, 26.5.

**Methyl 2-(4-(3-(2-fluoro-4-((2-(methylcarbamoyl)pyridin-4-yl)oxy)phenyl)ureido)phenyl)acetate (21l)**

Method D; purified by column chromatography on silica gel (EtOAc/CH_2_Cl_2_, 5:95 to 25:75) to afford **21l** (48%) as a brown solid; **^1^H NMR** (CDCl_3_, 500 MHz) 8.51 (1 H, s), 8.41 (1 H, d, *J* = 5.2 Hz), 8.32–8.28 (2 H, m), 7.95 (1 H, s), 7.60 (1 H, d, *J* = 2.3 Hz), 7.35 (2 H, d, *J* = 8.6 Hz), 7.16 (2 H, d, *J* = 8.6 Hz), 7.07 (1 H, dd, *J* = 5.4, 2.3 Hz), 6.84 (1 H, d, *J* = 9.2 Hz), 6.78 (1 H, dd, *J* = 10.9, 2.3 Hz), 3.69 (3 H, s), 3.57 (2 H, s), 3.01 (3 H, d, *J* = 5.2 Hz); **^13^C NMR** (CDCl_3_, 125 MHz) 172.7, 166.3, 165.3, 152.8, 152.2 (d, *J*_CF_ = 251.0 Hz), 151.4, 149.8, 147.3 (d, *J*_CF_ = 9.8 Hz), 137.9, 129.7, 128.2, 125.8 (d, *J*_CF_ = 9.8 Hz), 121.8, 119.6, 117.0, 115.1, 109.0, 108.2 (d, *J*_CF_ = 22.2 Hz), 52.1, 40.5, 26.4.

**4-(4-(3-(4-(1,1-Dioxido-3-oxo-1,2,5-thiadiazolidin-2-yl)-3-methylphenyl)ureido)-3-fluorophenoxy)-*N*-methylpicolinamide (CSR24)**

Method D; purified by column chromatography on silica gel (MeOH/CH_2_Cl_2_, 2:98 to 20:80) to afford **CSR24** (38%) as a brown solid; **^1^H NMR** (DMSO-*d_6_*, 500 MHz) 9.06 (1 H, s), 8.79 (1 H, s), 8.62 (1 H, s), 8.51 (1 H, d, *J* = 5.7 Hz), 8.24 (1 H, s), 7.42 (1 H, d, *J* = 2.3 Hz), 7.34–7.30 (3 H, m), 7.28 (1 H, s), 7.25 (1 H, d, *J* =2.3 Hz), 7.16 (1 H, dd, *J* = 5.2, 2.3 Hz), 7.05 (1 H, d, *J* = 7.4 Hz), 3.86 (2 H, s), 2.78 (3 H, d, *J* = 4.6 Hz), 2.29 (3 H, s); **HRMS** (ESI-TOF) *m/z*: [M – H]^–^ calculated for C_23_H_20_FN_6_O_6_S 527.1155; found: 527.1174.

**4-(4-(3-(3-(Cyanomethyl)phenyl)ureido)-3-fluorophenoxy)-*N*-methylpicolinamide (CSR25)**

Method D; purified by column chromatography on silica gel (EtOAc/CH_2_Cl_2_, 5:95 to 30:70) to afford **CSR25** (46%) as a white solid; **^1^H NMR** (DMSO-*d_6_*, 500 MHz) 9.21 (1 H, s), 8.79 (1 H, d, *J* = 5.2 Hz), 8.64 (1 H, s), 8.51 (1 H, d, *J* = 5.7 Hz), 8.23 (1 H, t, *J* = 9.2 Hz), 7.52 (1 H, s), 7.40 (1 H, d, *J* = 2.3 Hz), 7.38 (1 H, d, *J* = 8.6 Hz), 7.34–7.29 (2 H, m), 7.17 (1 H, dd, *J* = 5.7, 2.9 Hz), 7.06 (1 H, d, *J* = 9.2 Hz), 6.96 (1 H, d, *J* = 7.4 Hz), 4.03 (2 H, s), 2.78 (3 H, d, *J* = 5.2 Hz); **^13^C NMR** (DMSO-*d_6_*, 125 MHz) 165.6, 163.7, 152.5, 152.3 (d, *J*_CF_ = 244.9 Hz), 152.1, 150.5, 147.5 (d, *J*_CF_ = 11.1 Hz), 139.9, 132.0, 129.6, 125.4 (d, *J*_CF_ = 11.1 Hz), 121.8, 121.7, 121.7, 119.2, 117.4, 117.2, 114.1, 109.1 (d, *J*_CF_ = 22.2 Hz), 108.9, 26.0, 22.5.

**4-(4-(3-(3-(2-Aminoethyl)phenyl)ureido)-3-fluorophenoxy)-*N*-methylpicolinamide (CSR27)**

To a solution of **CSR25** (10.3 mg, 0.02 mmol) in anhydrous MeOH (2 mL) was added 10% Pd/C (1 mg), and catalytic amount of conc. HCl. The mixture was stirred under a H_2_ atmosphere for 2 days. The catalyst was removed by filtration through a Celite pad, and the filtrate was concentrated. The residue was purified by column chromatography (MeOH/CH_2_Cl_2_, 10:90 to 20:80) to afford **CSR27** (10.0 mg, 99%) as a brown solid; **^1^H NMR** (DMSO-*d_6_*, 500 MHz) 9.77 (1 H, s), 9.00 (1 H, s), 8.79 (1 H, d, *J* = 5.2 Hz), 8.51 (1 H, d, *J* = 5.7 Hz), 8.20 (1 H, t, *J* = 8.6 Hz), 8.08 (2 H, br), 7.41–7.40 (2 H, m), 7.35 (1 H, d, *J* = 8.0 Hz), 7.30 (1 H, dd, *J* = 11.5, 2.9 Hz), 7.24 (1 H, t, *J* = 7.4 Hz), 7.16 (1 H, dd, *J* = 5.4, 2.3 Hz), 7.04 (1 H, d, *J* = 8.6 Hz), 6.87 (1 H, d, *J* = 7.4 Hz), 3.01–2.98 (2 H, m), 2.87–2.84 (2 H, m), 2.78 (3 H, t, *J* = 5.2 Hz); **^13^C NMR** (DMSO-*d_6_*, 125 MHz) 165.6, 163.7, 152.5, 152.4, 152.3 (d, *J*_CF_ = 246.1 Hz), 150.5, 147.4 (d, *J*_CF_ = 11.1 Hz), 139.9, 138.0, 129.1, 125.6, 122.2, 121.9, 118.2, 117.0, 116.5, 114.1, 109.0 (d, *J*_CF_ = 27.1 Hz), 108.9, 33.1, 26.0.

**Methyl 2-(3-(3-(2-fluoro-4-((2-(methylcarbamoyl)pyridin-4-yl)oxy)phenyl)ureido)phenyl)acetate (CSR30)**

Method D; purified by column chromatography on silica gel (EtOAc/CH_2_Cl_2_, 5:95 to 25:75) to afford **CSR30** (41%) as a yellow solid; **^1^H NMR** (CDCl_3_, 600 MHz) 8.55 (1 H, s), 8.41 (1 H, d, *J* = 5.5 Hz), 8.33–8.28 (2 H, m), 7.94 (1 H, s), 7.61 (1 H, d, *J* = 2.1 Hz), 7.47 (1 H, s), 7.23–7.18 (2 H, m), 7.07 (1 H, dd, *J* = 5.5, 2.8 Hz), 6.91 (1 H, d, *J* = 7.6 Hz), 6.84 (1 H, d, *J* = 8.9 Hz), 6.79 (1 H, dd, *J* = 11.3, 2.8 Hz), 3.68 (3 H, s), 3.59 (3 H, s), 3.02 (3 H, d, *J* = 4.8 Hz); **^13^C NMR** (CDCl_3_, 150 MHz) 172.6, 166.3, 165.3, 152.8, 152.2 (d, *J*_CF_ = 245.4 Hz), 151.5, 149.8, 147.3, (d, *J*_CF_ = 10.4 Hz), 139.2, 134.6, 129.0, 125.7 (d, *J*_CF_ = 10.4 Hz), 123.7, 121.8, 120.2, 118.1, 116.9, 115.0, 109.0, 108.2 (d, *J*_CF_ = 22.2 Hz), 52.1, 41.1, 26.4.

**Methyl 2-((2-fluoro-4-(3-(2-fluoro-4-((2-(methylcarbamoyl)pyridin-4-yl)oxy)phenyl)ureido)phenyl)thio)acetate (CSR36)**

Method D; purified by column chromatography on silica gel (EtOAc/CH_2_Cl_2_, 5:95 to 25:75) to afford **CSR36** (61%) as a brown solid; **^1^H NMR** (CDCl_3_, 500 MHz) 8.79 (1 H, s), 8.46 (1 H, d, *J* = 5.7 Hz), 8.42 (2 H, m), 7.98 (1 H, s), 7.57 (1 H, d, *J* = 2.3 Hz), 7.52 (1 H, dd, *J* = 11.7, 2.3 Hz), 7.38 (1 H, t, *J* = 8.0 Hz), 7.17 (1 H, dd, *J* = 5.4, 2.3 Hz), 7.06 (1 H, dd, *J* = 8.6, 1.7 Hz), 6.91 (1 H, d, *J* = 9.2 Hz), 6.81 (1 H, dd, *J* = 10.9, 2.3 Hz), 3.68 (3 H, s), 3.54 (2 H, s), 3.03 (3 H, d, *J* = 5.2 Hz); **^13^C NMR** (CDCl_3_, 100 MHz) 170.4, 166.3, 165.5, 162.8 (d, *J*_CF_ = 245.5 Hz), 152.2, 152.0 (d, *J*_CF_ = 246.5 Hz), 151.1, 149.9, 147.2 (d, *J*_CF_ = 9.8 Hz), 141.8 (d, *J*_CF_ = 11.7 Hz), 135.3, 125.7 (d, *J*_CF_ = 10.8 Hz), 121.5, 117.1, 115.6, 114.4, 112.7 (d, *J*_CF_ = 18.6 Hz), 108.5, 108.2 (d, *J*_CF_ = 22.5 Hz), 106.4 (d, *J*_CF_ = 28.4 Hz), 52.5, 36.8, 26.5.

**General Procedure for the Preparation of (3-(2-fluoro-4-((2-(methylcarbamoyl)pyridin-4-yl)oxy)phenyl)ureido)carboxylic acids; 4-(3-(2-Fluoro-4-((2-(methyl carbamoyl)pyridin-4-yl)oxy)phenyl)ureido)benzoic acid (CSR1), Method E.**

To a solution of **21i** (18.8 mg, 0.04 mmol) in THF (3 mL) and water (1 mL) was added lithium hydroxide (2.9 mg, 0.12 mmol). The reaction was put into a pre-heated oil bath (60 ºC) and stirred for 18 h. After being quenched by the addition of 1 N HCl and water, the aqueous layer was extracted with EtOAc (2 × 15 mL). The combined organic extracts were washed with brine, dried over anhydrous Na_2_SO_4_, filtered, and concentrated. The residue was purified by column chromatography on silica gel (MeOH/CH_2_Cl_2_, 5:95 to 10:90) to afford **CSR1** (14.2 mg, 78%) as a white solid; **^1^H NMR** (DMSO-*d_6_*, 500 MHz) 12.63 (1 H, br), 9.47 (1 H, s), 8.80–8.77 (2 H, m), 8.52 (1 H, d, *J* = 5.2 Hz), 8.21 (1 H, t, *J* = 9.2 Hz), 7.88 (1 H, d, *J* = 8.6 Hz), 7.57 (1 H, d, *J* = 8.6 Hz), 7.41 (1 H, d, *J* = 2.9 Hz), 7.34 (1 H, dd, *J* = 11.5, 2.3 Hz), 7.18 (1 H, dd, *J* = 5.7, 2.9 Hz), 7.07 (1 H, d, *J* = 9.2 Hz), 2.78 (3 H, d, *J* = 5.2 Hz); **^13^C NMR** (DMSO-*d_6_*, 125 MHz) 167.0, 165.5, 163.7, 152.5, 152.4 (d, *J*_CF_ = 244.9 Hz), 152.0, 150.0, 147.8 (d, *J*_CF_ = 9.8 Hz), 143.6, 130.6, 125.2 (d, *J*_CF_ = 11.1 Hz), 124.0, 122.0, 117.2, 114.1, 109.1 (d, *J*_CF_ = 22.2 Hz), 108.9, 26.0; **HRMS** (DART-TOF) *m/z*: [M + H]^+^ calculated for C_21_H_18_FN_4_O_5_ 425.1256; found 425.1290.

**3-(3-(2-Fluoro-4-((2-(methylcarbamoyl)pyridin-4-yl)oxy)phenyl)ureido)benzoic acid (CSR2)**

Method E; purified by column chromatography on silica gel (MeOH/CH_2_Cl_2_, 5:95 to 10:90) to afford **CSR2** (61%) as a white solid; **^1^H NMR** (DMSO-*d_6_*, 500 MHz) 12.94 (1 H, br), 9.29 (1 H, s), 8.79 (1 H, d, *J* = 5.2 Hz), 8.65 (1 H, d, *J* = 1.7 Hz), 8.52 (1 H, d, *J* = 5.2 Hz), 8.22 (1 H, t, *J* = 9.2 Hz), 8.14 (1 H, d, *J* = 1.7 Hz), 7.62 (1 H, dd, *J* = 8.3, 1.2 Hz), 7.56 (1 H, d, *J* = 8.0 Hz), 7.43–7.40 (2 H, m), 7.33 (1 H, dd, *J* = 11.7, 2.3 Hz), 7.17 (1 H, dd, *J* = 5.4, 2.3 Hz), 7.06 (1 H, dd, *J* = 9.2, 1.7 Hz), 2.78 (3 H, d, *J* = 4.6 Hz); **^13^C NMR** (DMSO-*d_6_*, 125 MHz) 167.2, 165.6, 163.7, 152.5, 152.4 (d, *J*_CF_ = 245.9 Hz), 152.2, 150.5, 147.6 (d, *J*_CF_ = 10.8 Hz), 139.6, 131.4, 129.1, 125.4 (d, *J*_CF_ = 10.8 Hz), 123.0, 122.3, 121.9, 118.7, 117.2, 114.1, 109.1 (d, *J*_CF_ = 21.6 Hz), 108.9, 26.0; **HRMS** (DART-TOF) *m/z*: [M + H]^+^ calculated for C_21_H_18_FN_4_O_5_ 425.1256; found 425.1289.

**2-(3-(3-(2-Fluoro-4-((2-(methylcarbamoyl)pyridin-4-yl)oxy)phenyl)ureido)phenyl)-2-methylpropanoic acid (CSR29)**

Method E; purified by column chromatography on silica gel (MeOH/CH_2_Cl_2_, 2.5:97.5 to 5:95) to afford **CSR29** (72%) as a white solid; **^1^H NMR** (DMSO-*d_6_*, 500 MHz) 9.21 (1 H, s), 8.79 (1 H, d, *J* = 4.6 Hz), 8.64 (1 H, s), 8.51 (1 H, d, *J* = 5.7 Hz), 8.21 (1 H, t, *J* = 9.2 Hz), 7.45 (1 H, s), 7.41 (1 H, d, *J* = 2.3 Hz), 7.36–7.30 (2 H, m), 7.24 (1 H, t, *J* = 7.4 Hz), 7.17 (1 H, dd, *J* = 5.7, 2.3 Hz), 7.04 (1 H, dd, *J* = 8.6, 1.7 Hz), 6.97 (1 H, d, *J* = 7.4 Hz), 2.78 (3 H, d, *J* = 4.6 Hz), 1.45 (6 H, s); **^13^C NMR** (DMSO-*d_6_*, 125 MHz) 177.5, 165.6, 163.7, 152.5, 152.3 (d, *J*_CF_ = 248.6 Hz), 152.2, 150.5, 147.4 (d, *J*_CF_ = 11.1 Hz), 139.3, 128.7, 125.6 (d, *J*_CF_ = 9.8 Hz), 121.8, 119.3, 117.1, 117.0, 116.2, 115.5, 114.1, 109.0 (d, *J*_CF_ = 22.2 Hz), 108.9, 45.8, 26.4, 26.0.

**2-(3-(3-(2-Fluoro-4-((2-(methylcarbamoyl)pyridin-4-yl)oxy)phenyl)ureido)phenyl)acetic acid (CSR31)**

Method E; purified by column chromatography on silica gel (MeOH/CH_2_Cl_2_, 5:95) to afford **CSR31** (74%) as a white solid; **^1^H NMR** (DMSO-*d_6_*, 500 MHz) 9.18 (1 H, s), 8.80 (1 H, d, *J* = 5.2 Hz), 8.69 (1 H, s), 8.51 (1 H, d, *J* = 5.7 Hz), 8.22 (1 H, t, *J* = 9.2 Hz), 7.41 (1 H, d, *J* = 2.9 Hz), 7.37–7.30 (3 H, m), 7.22 (1 H, t, *J* = 8.0 Hz), 7.17 (1 H, dd, *J* = 5.7, 2.3 Hz), 7.05 (1 H, dd, *J* = 8.9, 1.7 Hz), 6.87 (1 H, d, *J* = 8.0 Hz), 3.51 (2 H, s), 2.78 (1 H, d, *J* = 4.6 Hz); **^13^C NMR** (DMSO-*d_6_*, 125 MHz) 172.8, 165.6, 163.7, 152.5, 152.3 (d, *J*_CF_ = 242.4 Hz), 152.2, 150.5, 147.4 (d, *J*_CF_ = 8.6 Hz), 139.4, 135.9, 128.8, 125.6 (d, *J*_CF_ = 9.8 Hz), 123.2, 121.8, 119.0, 117.1, 116.4, 114.0, 109.1 (d, *J*_CF_ = 22.2 Hz), 108.9, 41.1, 26.0.

**2-((3-(3-(2-Fluoro-4-((2-(methylcarbamoyl)pyridin-4-yl)oxy)phenyl)ureido)phenyl)thio)acetic acid (CSR32)**

Method E; purified by column chromatography on silica gel (MeOH/CH_2_Cl_2_, 5:95) to afford **CSR32** (75%) as a yellow solid; **^1^H NMR** (DMSO-*d_6_*, 500 MHz) 9.23 (1 H, s), 8.80 (1 H, d, *J* = 4.6 Hz), 8.75 (1 H, s), 8.51 (1 H, d, *J* = 5.2 Hz), 8.20 (1 H, t, *J* = 8.6 Hz), 7.46 (1 H, s), 7.41 (1 H, d, *J* = 2.9 Hz), 7.32 (1 H, dd, *J* = 11.7, 2.3 Hz), 7.24–7.20 (2 H, m), 7.17 (1 H, dd, *J* = 5.4, 2.3 Hz), 7.05 (1 H, dd, *J* = 8.9, 1.7 Hz), 6.93 (1 H, d, *J* = 6.9 Hz), 3.75 (2 H, s), 2.78 (3 H, d, *J* = 4.6 Hz); **^13^C NMR** (DMSO-*d_6_*, 125 MHz) 170.6, 165.6, 163.7, 152.5, 152.4 (d, *J*_CF_ = 244.9 Hz), 152.2, 150.5, 147.6 (d, *J*_CF_ = 9.8 Hz), 140.0, 136.8, 129.4, 125.4 (d, *J*_CF_ = 11.1 Hz), 122.0, 121.0, 117.1, 116.6, 115.5, 114.1, 109.1 (d, *J*_CF_ = 22.2 Hz), 108.9, 35.3, 26.0.

**2-(4-(3-(2-Fluoro-4-((2-(methylcarbamoyl)pyridin-4-yl)oxy)phenyl)ureido)phenyl)acetic acid (CSR33)**

Method E; purified by column chromatography on silica gel (MeOH/CH_2_Cl_2_, 2.5:97.5 to 5:95) to afford **CSR33** (74%) as a white solid; **^1^H NMR** (DMSO-*d_6_*, 400 MHz) 9.13 (1 H, s), 8.80 (1 H, d, *J* = 4.6 Hz), 8.68 (1 H, s), 8.51 (1 H, d, *J* = 5.5 Hz), 8.22 (1 H, t, *J* = 9.2 Hz), 7.41–7.38 (3 H, m), 7.32 (1 H, dd, *J* = 11.9, 2.8 Hz), 7.18–7.16 (3 H, m), 7.05 (1 H, d, *J* = 8.7 Hz), 3.48 (2 H, s), 2.78 (3 H, d, *J* = 4.6 Hz); **^13^C NMR*** (DMSO-*d_6_*, 100 MHz) 173.0, 165.6, 163.7, 152.5, 152.3, 152.2 (d, *J*_CF_ = 246.5 Hz), 150.5, 147.4 (d, *J*_CF_ = 10.8 Hz), 137.9, 129.8, 125.7 (d, *J*_CF_ = 14.7 Hz), 121.7, 118.1, 117.2, 114.1, 109.1 (d, *J*_CF_ = 20.5 Hz), 108.9, 26.0.

*One of ^13^C NMR peak was hidden in DMSO-*d_6_* peaks due to low sample concentration.

**2-((4-(3-(2-Fluoro-4-((2-(methylcarbamoyl)pyridin-4-yl)oxy)phenyl)ureido)phenyl)thio)acetic acid (CSR34)**

Method E; purified by column chromatography on silica gel (MeOH/CH_2_Cl_2_, 10:90 to 20:80) to afford **CSR34** (86%) as a brown solid; **^1^H NMR** (DMSO-*d_6_*, 500 MHz) 12.7 (1 H, br), 9.19 (1 H, s), 8.80 (1 H, d, *J* = 5.2 Hz), 8.68 (1 H, s), 8.51 (1 H, d, *J* = 5.7 Hz), 8.20 (1 H, t, *J* = 9.2 Hz), 7.43–7.40 (3 H, m), 7.33–7.31 (3 H, m), 7.17 (1 H, dd, *J* = 5.4, 2.3 Hz), 7.05 (1 H, d, *J* = 7.4 Hz), 3.67 (2 H, s), 2.78 (3 H, 4.6 Hz); **^13^C NMR** (DMSO-*d_6_*, 125 MHz) 170.8, 165.6, 163.7, 152.5, 152.3 (d, *J*_CF_ = 230.1), 152.1, 150.5, 147.5 (d, *J*_CF_ = 16.0 Hz), 138.3, 130.4, 125.5 (d, *J*_CF_ = 11.1 Hz), 121.8, 118.7, 117.0, 114.1, 109.1 (d, *J*_CF_ = 22.2 Hz), 108.9, 36.9, 26.0.

**2-((2-Fluoro-4-(3-(2-fluoro-4-((2-(methylcarbamoyl)pyridin-4-yl)oxy)phenyl)ureido)phenyl)thio)acetic acid (CSR35)**

Method E; purified by column chromatography on silica gel (MeOH/CH_2_Cl_2_, 5:95 to 15:85) to afford **CSR35 (**98%) as a yellow solid; **^1^H NMR** (DMSO-*d_6_*, 500 MHz) 12.7 (1 H, br), 9.37 (1 H, s), 8.81–8.78 (1 H, m), 8.73 (1 H, d, *J* = 1.7 Hz), 8.52 (1 H, d, *J* = 5.7 Hz), 8.18 (1 H, t, *J* = 8.6 Hz), 7.55 (1 H, dd, *J* = 12.3, 2.3 Hz), 7.42–7.38 (2 H, m), 7.33 (1 H, dd, *J* = 11.7, 2.3 Hz), 7.18 (1 H, dd, *J* = 5.4, 2.3 Hz), 7.11 (1 H, dd, *J* = 8.6, 2.3 Hz), 7.06 (1 H, dd, *J* = 8.6, 1.7 Hz), 3.64 (2 H, s), 2.78 (3 H, d, *J* = 4.6 Hz); **^13^C NMR** (DMSO-*d_6_*, 125 MHz) 170.4, 165.5, 163.7, 161.0 (d, *J*_CF_ = 241.2 Hz), 152.5, 152.5 (d, *J*_CF_ = 244.9 Hz), 150.5, 147.8 (d, *J*_CF_ = 11.1 Hz) 140.7 (d, *J*_CF_ = 11.1 Hz), 133.3, 125.1 (d, *J*_CF_ = 11.1 Hz), 122.1, 117.2, 114.4, 114.1, 113.3, 113.2, 109.1 (d, *J*_CF_ = 22.2 Hz), 108.9, 105.2 (d, *J*_CF_ = 28.3 Hz), 35.8, 26.0.

**Receptor interacting protein kinase 2 (RIPK2) and KDR (VEGFR2) enzyme assays**

Expression and purification of recombinant human His6-RIPK2 and His6-RIRK2 R171C, residues 8-317, was performed as previously described (for details see: Canning P, Ruan Q, Schwerd T, et al. Inflammatory Signaling by NOD-RIPK2 Is Inhibited by Clinically Relevant Type II Kinase Inhibitors. Chem Biol. 2015;22(9): 1174–1184). Commercial KDR enzyme was used (SignalChem). For ADPGlo (Promega) assays, 5 ng KDR or 10 ng of RIPK2 was diluted in reaction buffer (40 mM Tris-HCl pH 7.5, 20 mM MgCl_2_, 0.05 mM DTT, 0.01% BSA) supplemented with 50 μM ATP and 1 μg/reaction substrates RS repeat peptide (SignalChem, for RIPK2) or poly-E4Y1 (Sigma, for KDR) and 8-point dose range of inhibitors. Reactions were performed at room temperature for 2 hours. Reactions were performed in 5 μL total volume (5% final concentration of DMSO) and stopped by addition of 5 μL of ADPGlo reagent for 40 min at room temperature. Luminescent signal was generated by addition of 10 μL of kinase detection reagent for 30 minutes at room temperature and determined using Victor3V platereader (Perkin Elmer). Specific signal was calculated by subtracting values in the wells without protein and inhibitor from the values in the test wells. The percent inhibition at a specified concentration is determined or IC_50_ values are calculated based on a dose range of inhibitor concentrations using non-linear regression in GraphPad Prism software. IC_50_ values are based on four independent determinations.

**Table S1**. Data collection and refinement statistics for RIPK2•**CSR35** (PDB ID: 6ES0).

| **Wavelength (Å)** | 0.9163 |
| --- | --- |
| **Resolution range (Å)** | 71.74 - 2.38 (2.47 - 2.38) |
| **Space group** | P2_1_2_1_ 2_1_ |
| **Unit cell dimensions - a,b,c (Å) α,β,γ (°)** | 61.98 83.74 139.1 90 90 90 |
| **Total reflections** | 135261 (12861) |
| **Unique reflections** | 29591 (2906) |
| **Multiplicity** | 4.6 (4.4) |
| **Completeness (%)** | 99.41 (99.66) |
| **Mean I/sigma(I)** | 16.05 (3.81) |
| **Wilson B-factor (Å^2^)** | 34.57 |
| **R-merge** | 0.080 (0.399) |
| **R-meas** | 0.091 (0.453) |
| **R-pim** | 0.042 (0.211) |
| **CC1/2** | 0.998 (0.689) |
| **CC*** | 1 (0.903) |
| **Reflections used in refinement** | 29591 (2906) |
| **Reflections used for R-free** | 1480 (145) |
| **R-work** | 0.214 (0.291) |
| **R-free** | 0.258 (0.345) |
| **Number of non-hydrogen atoms** | 4901 |
| **macromolecules** | 4638 |
| **ligands** | 68 |
| **solvent** | 195 |
| **Protein residues** | 575 |
| **RMS (bond lengths, Å)** | 0.003 |
| **RMS (bond angles, °)** | 0.59 |
| **Ramachandran favored (%)** | 98.05 |
| **Ramachandran allowed (%)** | 1.95 |
| **Ramachandran outliers (%)** | 0.00 |
| **Average B-factor (Å^2^)** | 39.76 |
| **macromolecules (Å^2^)** | 39.82 |
| **ligands (Å^2^)** | 32.89 |
| **solvent (Å^2^)** | 40.80 |
